# Compositional heterogeneity confers selective advantage to model protocellular membranes during the origins of cellular life

**DOI:** 10.1038/s41598-020-61372-w

**Published:** 2020-03-11

**Authors:** Susovan Sarkar, Shikha Dagar, Ajay Verma, Sudha Rajamani

**Affiliations:** Department of Biology, Indian Institute of Science Education and Research, Pune, 411008 India

**Keywords:** Lipids, Biochemistry, Chemical biology, Membranes, Molecular evolution, Origin of life

## Abstract

Protocellular membranes are thought to be composed of mixtures of single chain amphiphiles, such as fatty acids and their derivatives, moieties that would have been part of the complex prebiotic chemical landscape. The composition and physico-chemical properties of these prebiological membranes would have been significantly affected and regulated by their environment. In this study, pertinent properties were systematically characterized, under early Earth conditions. Two different fatty acids were mixed with their respective alcohol and/or glycerol monoester derivatives to generate combinations of binary and tertiary membrane systems. Their properties were then evaluated as a function of multiple factors including their stability under varying pH, varying Mg^2+^ ion concentrations, dilution regimes, and their permeability to calcein. Our results demonstrate how environmental constraints would have acted as important prebiotic selection pressures to shape the evolution of prebiological membranes. The study also illustrates that compositionally diverse membrane systems are more stable and robust to multiple selection pressures, thereby making them more suitable for supporting protocellular life.

## Introduction

The earliest forms of cellular life are considered to be entities that comprised of dynamic chemical reactions, encapsulated within amphiphilic compartments^1,2^. Unlike the contemporary biological membranes model protocellular membranes are thought to have been relatively simpler and composed of single chain amphiphiles (SCAs)^3^. These SCAs could have come about on the early Earth either by endogenous synthesis, in the form of Fisher-Tropsch Type (FTT) reactions, or via exogenous delivery^4,5^. In this context, fatty acids and their derivatives have been predominantly studied for their plausible role as early compartments^3,6^. Fatty acids are known to possess high critical vesicular concentrations (CVCs), the concentration at which the monomers assemble into higher ordered structures like vesicles^7^. Such high CVC requirement poses significant obstacles towards their self- assembly under prebiotic scenarios, wherein meeting this high concentration prerequisite would have been difficult^8,9^. The pH of certain terrestrial hydrothermal pools of the early Earth is hypothesized to be neutral to alkaline^10^ which can drive prebiotically pertinent reactions, including formose reaction^11^, polymerization of non-activated amino acids^12^, and non-canonical nucleoside or nucleotide formation^13^. However fatty acid monomers can assemble only in a narrow pH regime, near to their pKa^6,14^.

Given this scenario the coexistence of the aforementioned reactions and model protocellular membranes would have been really challenging. Moreover, fatty acids are also cation sensitive moieties^15^. On the contrary, RNA molecules, which are thought to be the first biomolecules to have emerged, require divalent cations in order to efficiently replicate and carry out catalytic functions^16–18^. Such divalent cation concentrations are not compatible with fatty acid membranes^15,19^. This poses an imminent question of how RNA replicators could have coexisted with fatty acid based membranes. Nonetheless, fatty acid membranes are in dynamic equilibrium hence can facilitate the permeation of polar molecules better^20^, which is an essential requirement for protocells as it allows for the exchange of matter with its environment. In this regard, the self-assembly property, permeability and membrane integrity in different environmental conditions of such SCAs need to be systematically explored. Thus far, studies have predominantly focused on delineating one, or up to two of the aforesaid parameters at any given time^3,6–9,19,21^. However, the aforementioned conditions would have acted in concert as a combination of prebiotic selection pressures, shaping the evolutionary landscape of prebiological membranes. Previous studies have suggested that the lack of membrane stability can be counterbalanced by increasing membrane complexity and can facilitate formation of lipid catalytic networks^22^. It is known that addition of long chain alcohols with fatty acids, decreases the CVC of the resultant binary systems^8^, and also confers stability to the vesicles at alkaline pH^23^. Previous studies have also showed that binary systems of fatty acid and its glycerol monoester are more resistant to soluble monovalent and divalent cations^19,24^. However, the mechanism underlying this increase in stability towards divalent cations, in the presence of derivatives, is not clearly understood. Although insightful, the aforementioned studies predominantly looked at binary membrane systems. Given the complex nature of the prebiotic soup, and the niche parameters, it would be worthwhile to complexify the starting mix, to better understand how membrane related processes would have advent under ‘prebiotically realistic’ conditions. In this context, a membrane system composed of decanoic acid, decanol and glycerol mono-decanoate, is the only tertiary system that has been explored thus far, in terms of its thermostability and permeability^25,26^. In order to gain a deeper understanding of how compositional complexity would impinge on a membrane system’s survivability, especially under multiple prebiotic selection pressures, we set out to characterize tertiary membrane systems of selected SCAs. In the present study, fatty acids of two different chain lengths, i.e. oleic acid (OA, C18) and undecylenic acid (UDA, C11), were mixed with their corresponding alcohol and/or glycerol monoester derivatives in varying ratio, and used as a proxy for mixed membrane systems. Fatty alcohols and glycerol monoester derivatives were chosen for further experimentation because of their prebiotic relevance^4,9^. Binary membrane systems containing fatty acid with either the fatty alcohol or the glycerol monoester derivative, and tertiary systems containing all the three components, were explored for each of the chain lengths. The prebiotically relevant physical parameters that were characterized include the formation of model protocellular membranes at alkaline pH, their CVC, ionic stability and the permeability of the said systems. Our results show that the mixed membrane systems are indeed more stable, and robust under diverse environmental conditions. Therefore, these would have been more suitable to support protocellular life forms. Our results also illustrate that the head groups of the SCAs play an important role in stabilizing the membrane under specific selection conditions. Systems containing different derivatives possess different survival rates when subjected to a specific selection pressure. Given this interesting finding, we also attempted to delineate the contribution of individual head-groups, and the plausible mechanism that might be involved in stabilizing the protomembrane systems. An important result of this study clearly demonstrates that the tertiary system being most complex would have possessed the best chance at survival when subjected to multiple selection pressures (MSP). The overall outcome illustrates that the evolution of protomembranes would have been shaped, both, by their compositional heterogeneity, and the niche parameters (selection conditions) that these systems were subjected to. This work, thus, has implications for discerning the emergence of mixed membrane systems, and highlights the need to factor prebiotically realistic conditions, to better understand how they would have impinged on the evolutionary landscape of prebiotic membranes.

## Results

### Formation of vesicle under alkaline pH regimes

All the experiments described in this present study were conducted using binary and tertiary mixed membrane systems of C11 and C18 fatty acids. The binary systems based on the undecylenic acid (UDA, C11) fatty acid system were prepared by mixing undecylenic acid (UDA) with either glyceryl 1-undecylenate (UDG) or undecylenyl alcohol (UDOH). Similarly, the oleic acid based (OA, C18) binary systems were prepared by mixing oleic acid (OA) either with glycerol 1-monooleate (GMO), or the oleyl alcohol (OOH) derivative. The ratio of fatty acid to its derivatives was fixed to either 4:1 or 2:1. The tertiary systems for both the chain lengths were prepared by mixing all the three respective components together, i.e. UDA,UDG and UDOH, and OA, GMO and OOH, in ratios of 2:1:1, 4:1:1 and 6:1:1, respectively. Fig. S1 in the supporting information summarizes the structures of the aforesaid amphiphiles used in this study.

**Figure 1:**
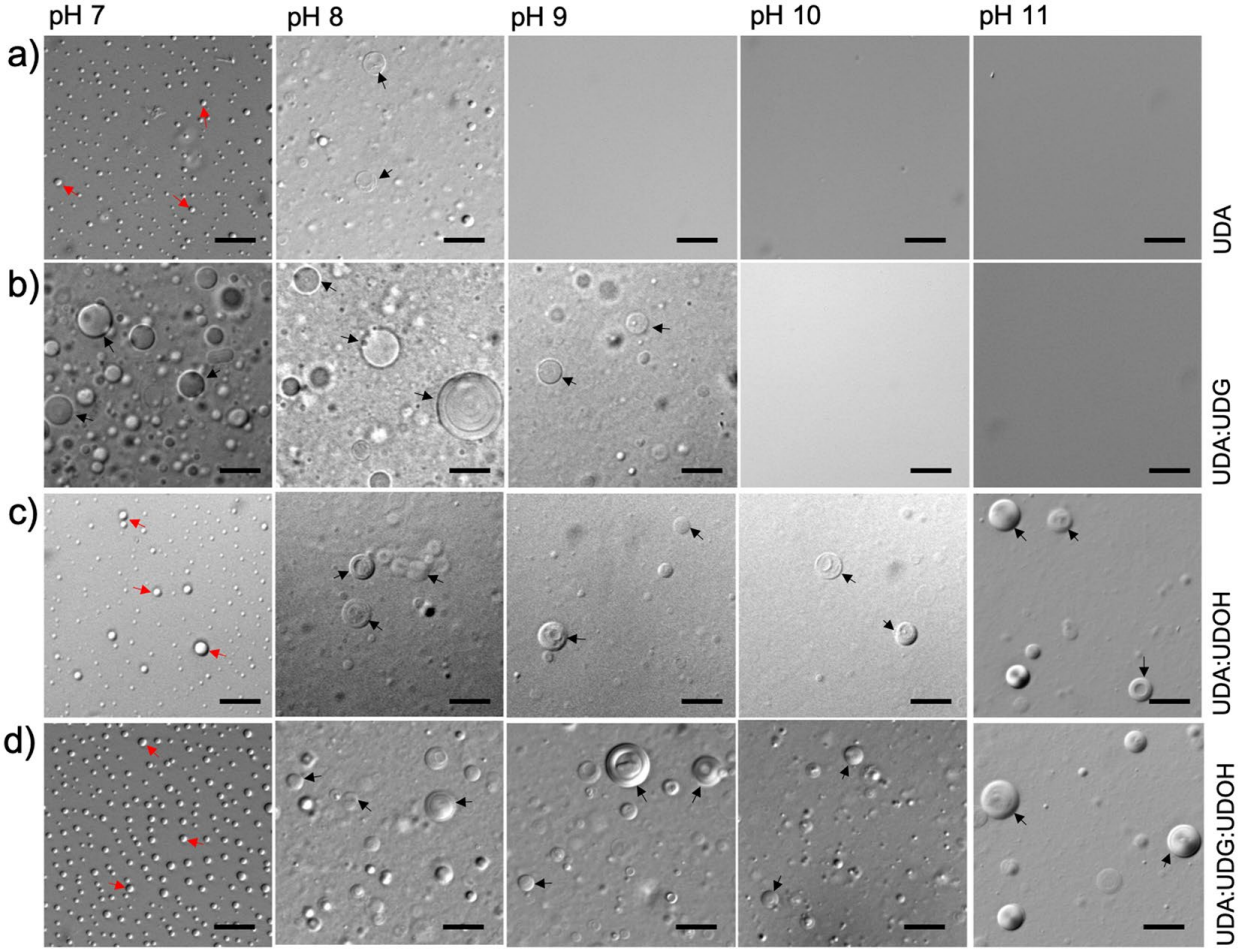
Formation of membrane under alkaline pH regimes. Microscopic analysis of C11 based membrane systems. These images demonstrate the formation of vesicles and oil droplets depending on the pH of the surrounding environment. Panels (**a**–**d**) show the four different C11 based membrane systems. The ratio of fatty acid to its respective glycerol monoester and/or alcohol was maintained at 2:1. The black and red arrows indicate vesicles and oil droplets, respectively. The scale bar in all the images is 10 microns. UDA, undecylenic acid; UDG, glyceryl 1-undecylenate; UDOH, undecylenyl alcohol.

**Figure 2:**
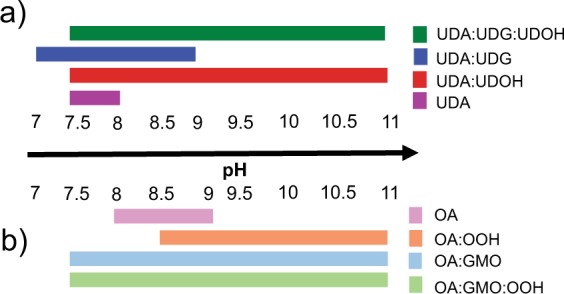
Summary of membrane formation ability under alkaline pH regimes. The illustration showing the ability of the different membrane systems to form vesicles over varying pH regimes. The ability of a system to form vesicles over the range of pH is represented by the colored horizontal bars. Panels (**a,b**) represent the UDA and OA based mixed systems, respectively. The ratio of fatty acid to its respective glycerol monoester and/or alcohol was maintained at 2:1. UDA, undecylenic acid; UDG, glyceryl 1-undecylenate; UDOH, undecylenyl alcohol; OA, oleic acid; GMO, glycerol 1-monooleate; OOH, oleyl alcohol.

The vesicle forming ability of the different membrane systems were evaluated from pH 7 to 11, at room temperature. It was observed that both the fatty alcohol and glycerol monoester derivatives could indeed stabilize the fatty acid vesicles over a wide range of pH. Absorption measurement of the suspension at 400 nm indicated that UDA alone could form vesicles from pH 7.5 to 8 (Supplementary Fig. S6), which was also confirmed using microscopy (Fig. 1). The binary mixed UDA:UDG (2:1) system formed vesicles from pH 7 to 9, above which micelle formation occurred that resulted in a decrease in the turbidity values. On the other hand, the UDA:UDOH (2:1) binary system, formed vesicles from pH 7.5 to 11 (Fig. 1). When the ratio of fatty acid to derivative was changed to 4:1, it was observed that the UDA:UDG (4:1) and the UDA:UDOH (4:1) binary systems assemble into vesicles from pH 7.5 to 8.5, and from pH 7.5 to 9, respectively (Supplementary Figs. S2 and S3). The tertiary systems of UDA:UDG:UDOH in 4:1:1 and 2:1:1 ratios were able to form vesicles from pH 7.5 to 11. The tertiary system of UDA:UDG:UDOH in 6:1:1 ratio formed vesicles from pH 7.5 to 9.5, as shown in Supplementary Fig. S2. As for the OA based systems, the OA itself formed vesicles from pH 8 to 9 (Supplementary Fig. S4). Below pH 8, large oil droplets were observed. The OA:OOH (2:1) binary system could form vesicles from pH 8.5 to 11. However, below 8.5, it assembled into large droplets. In the case of the OA:GMO (2:1) binary system and the tertiary system of OA:GMO:OOH in 4:1:1 ratio, vesicles were observed over a wide range of pH starting from 7.5, up to pH 11 (Supplementary Figs. S2 and S4). The binary mixed system OA:GMO (4:1) and OA:OOH (4:1) formed vesicles from pH 7.5 to 10 and pH 8.5 to 11, respectively (Supplementary Figs. S2 and S5). In case of the tertiary systems, OA:GMO:OOH (2:1:1) and OA:GMO:OOH (6:1:1), vesicles were observed from pH 8 to 11 (Supplementary Figs. S2 and S5). Based on all these observations, eight different membrane systems were taken forward for further exploration because of their overall robustness. Four systems were chosen for each of the chain length systems, i.e. pure acid system, two binary systems composed of fatty acid mixed with either its corresponding alcohol or glycerol monoester in 2:1 ratio, and the tertiary mixed system which contains all the three components in 4:1:1 ratio (Fig. 2). This way, the overall ratio of fatty acid to derivative was fixed to 2:1 to compare across the membrane systems effectively, which also corroborates with the ratios that have been used in previous protocell membrane studies^25,26^.

### Self-assembly of model protocellular membrane systems

The fluorescence assay using 1,6-diphenyl-1,3,5-hexatriene (DPH) as a membrane probe revealed that the CVC of the pure UDA was around 35 mM (see the Supplementary section for further details). Upon mixing UDA with UDG, the CVC drastically decreased to 2 mM (Supplementary Fig. S7). It is pertinent to note that the lipid concentration for the mixed systems refers to the total lipid concentration, which takes into account the fatty acid and the respective derivatives used in the different mixed systems studied. For the binary system of UDA:UDOH (2:1), and the tertiary system of UDA:UDG:UDOH (4:1:1), the CVC was found to be around 4 and 2 mM, respectively (Supplementary Fig. S7). Microscopic analysis of all the systems confirmed the presence of vesicles (Supplementary Fig. S11). For the pure oleic acid (OA) system, the CVC was found to be around 0.09 mM. Upon adding either the GMO or the OOH derivative (2:1 ratio), the CVC decreased to about 0.02 and 0.06 mM, respectively. For the tertiary system of OA:GMO:OOH (4:1:1), the CVC was found to be around 0.02 mM (Supplementary Fig. S8). The presence of vesicles was also confirmed by measuring the Absorbance (scattering) of the suspension at 400 nm (Supplementary Figs. S9 and S10). All the experiments were performed at room temperature. On performing microscopy, vesicles were observed under the microscope only at a slightly higher concentration than what was expected from the fluorescence assay (as is summarized in Table 1). This could potentially stem from the fact that light microscopy is diffraction-limited thus missing out on vesicles that are smaller than 200 nm.

**Table 1:** Summary of the CVCs of all the different C18 and C11 based systems using three different assays.

Apparent CVC in mM			
System used	Fluorescence Assay	Microscopy	Turbidity Assay
UDA	35	42	40
UDA:UDOH	4	12	8
UDA:UDG	2	2	2
UDA:UDG:UDOH	2	2	3
OA	0.09	0.6	0.2
OA:OOH	0.06	0.1	0.1
OA:GMO	0.01	0.1	0.1
OA:GMO:OOH	0.02	0.05	0.08

Columns 2, 3, and 4 provide a comparison of the difference in the CVC estimation using fluorescence assay, microscopy and turbidity assay, respectively. The molar ratio of fatty acid to overall derivative was kept to 2:1. UDA, undecylenic acid; UDG, glyceryl 1-undecylenate; UDOH, undecylenyl alcohol; OA, oleic acid; GMO, glycerol 1-monooleate; OOH, oleyl alcohol.

### Stability of vesicles in the presence of Mg^2+^ ions

**Figure 3:**
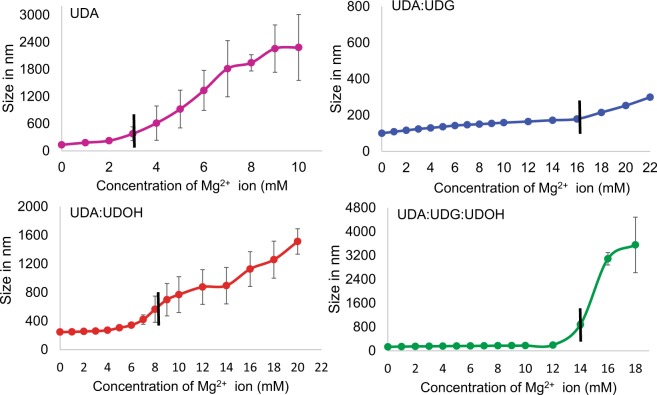
Membrane stability in presence of Mg^2^^+^ ions. Shows DLS measurements of C11 based membrane systems to determine the Mg^2+^ ion induced aggregate formation (crystalline aggregates and collapsed vesicles) concentration. The particle diameter (in nm) is plotted as a function of the added Mg^2+^ ion concentration. The vertical black dashed line indicates the Mg^2+^ ion induced aggregation formation concentration (Mg^2+^_AIC_). The ratio of fatty acid to its respective glycerol monoester and/or alcohol was maintained at 2:1. n = 3; error bars represent standard deviation (s.d.). UDA, undecylenic acid; UDG, glyceryl 1-undecylenate; UDOH, undecylenyl alcohol.

**Figure 4:**
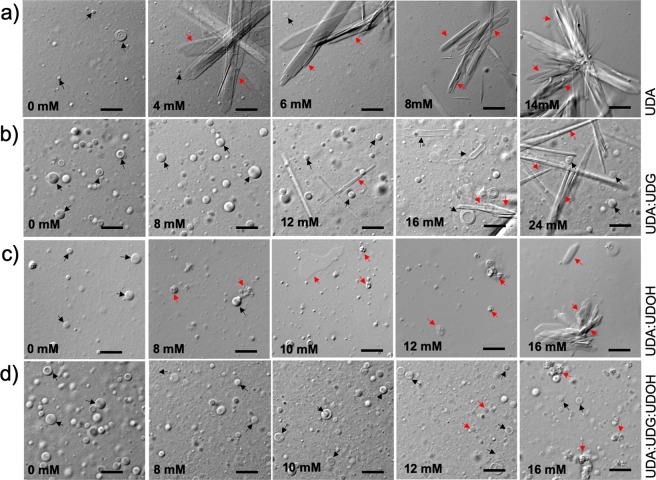
Microscopic analysis of model protocellular membrane stability in presence of Mg^2+^ ions. Mg^2+^ ion induced aggregate (crystalline aggregates and collapsed vesicles) forming propensities of all the four UDA based systems (panels **a**–**d**). From left to right, Mg^2+^ ions concentration was increased gradually by keeping the lipid concentration constant. In terms of cation sensitivity among the four systems, the following order is seen: UDA > UDA:UDOH > UDA:UDG:UDOH > UDA:UDG. The black and red arrows indicate vesicles and aggregates (crystalline aggregates and collapsed vesicles), respectively. The scale bar = 10 microns. The ratio of fatty acid to its respective glycerol monoester and/or alcohol was maintained at 2:1. UDA, undecylenic acid; UDG, glyceryl 1-undecylenate; UDOH, undecylenyl alcohol.

Dynamic Light Scattering (DLS) measurement was used to determine the stability of the vesicles in the presence of Mg^2+^ ions at room temperature (see Supplementary information for further details). The DLS analysis showed that the UDA:UDG (2:1) binary system was the most stable one, with a Mg^2+^ ion induced-aggregation concentration (Mg^2+^_AIC_) of 16 mM (see the method section for details), followed by the tertiary UDA:UDG:UDOH (4:1:1) and the binary UDA:UDOH (2:1) systems, with a Mg^2+^_AIC_ of 14 and 8 mM, respectively (Fig. 3). The pure UDA system was extremely labile to Mg^2+^ ions, where aggregation started even at Mg^2+^ ion concentration of as low as 3 mM (Fig. 3). On microscopic analysis, large crystalline aggregates (Mg-soap) were observed in the UDA system at 4 mM Mg^2+^ ion concentration, and no vesicles were observed in the solution beyond 8 mM Mg^2+^ concentration (see Fig. 4, panel a). However, in the case of the UDA:UDG system, crystalline aggregates and collapsed vesicles started appearing at 12 mM Mg^2+^ ion concentration, and vesicles persisted even in solutions containing a Mg^2+^ ion concentration of 24 mM (see Fig. 4, panel b). Between the UDA:UDOH and the UDA:UDG:UDOH systems, the latter seemed more stable, with vesicles being observed along with some crystalline aggregates (Mg-soap) and collapsed vesicles in the presence of 12 mM Mg^2+^ ions (see Fig. 4, panel d). However, only crystalline aggregates and collapsed vesicles were found in the UDA:UDOH system at 12 mM Mg^2+^ ion concentration under the microscope (see Fig. 4, panel c).

Among the four C18 based systems, the OA alone system was found to be the most sensitive, with a Mg^2+^_AIC_ of 3.5 mM (Supplementary Fig. S12). Interestingly the OA:GMO (2:1) was found to be the second most sensitive towards Mg^2+^ ions, with a Mg^2+^_AIC_ of 5 mM. Both, the binary OA:OOH (2:1) and the tertiary OA:GMO:OOH (4:1:1) systems showed an Mg^2+^_AIC_ of 6 mM (using DLS) (Supplementary Fig. S12). Similar observations were confirmed using microscopy (Supplementary Fig. S13). Table S1 in the supplementary information compares the Mg^2+^_AIC_ of all eight membrane systems across the two experimental methods that were used. Overall, the Mg^2+^_AIC_ of all the four C18 based systems were lower as compared to the C11 based systems. This could also be because of using a total of 2 mM lipid concentration for all the four C18 based systems, which is much lower than what was used for C11 based systems (60 mM for pure UDA and 20 mM for the mixed systems) due to its higher intrinsic CVC.

To further investigate if the resultant aggregates (collapsed vesicles and Mg-soap crystals) were indeed due to the presence of high concentrations of Mg^2+^ ions, EDTA (a chelating agent) was added to the preformed aggregates (see Supplementary method section for details). In the presence of EDTA, vesicles were reformed readily from the aggregates of collapsed vesicles and Mg-soap crystals in all four C11 based systems (Supplementary Fig. S14). Mg^2+^ ions were chelated and removed from the fatty acid-Mg^2+^ ion aggregates by EDTA, resulting in the reformation of vesicles.

### Permeability of model protocellular membranes

**Figure 5:**
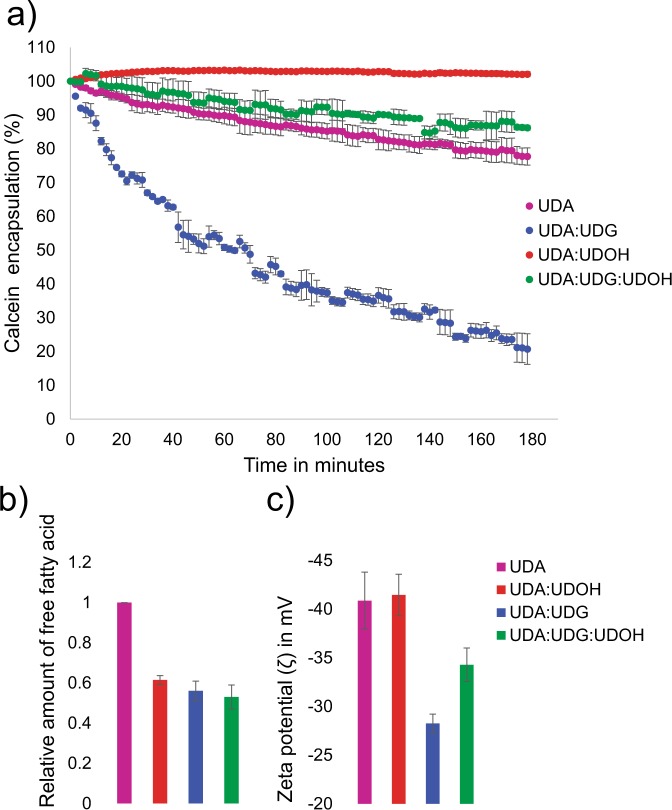
Membrane permeability as a function of its composition, LC-MS analysis for free acid quantification and zeta potential measurements. (**a**) Calcein leakage assay for the various UDA based systems. n = 4; error bars represent standard deviation (S.D). (**b**) shows the relative amount of free fatty acids in the solution as a function of the membrane composition. n = 6; error bars represent SD. The difference between the mean values for the pure UDA system with other three mixed systems is significant based on one-tailed student t test with a *p*-value < 0.05. (**c**) Indicates the zeta potential measurements of the different C11 based systems as a function of their composition. n = 5; error bars represent SD. The difference between the mean values for UDA with UDA:UDG and UDA:UDG:UDOH is significant based on one-tailed student t test with a *p*-value < 0.05. The ratio of fatty acid to its respective glycerol monoester and/or alcohol was maintained at 2:1.

C11 based systems were chosen to explore the effect of membrane composition heterogeneity on membrane permeability because of the intrinsic dynamicity that stems from having a smaller chain length in this case. Previous studies have looked at the permeability of pure OA and binary OA:GMO based system^24,25,27^. Calcein leakage assay was performed at room temperature to determine the permeability of the protocell membranes (see Experimental Section and Supplementary for more details). It was observed that when UDG was mixed with UDA, the permeability of this mixed binary membrane system increased drastically, in comparison to the pure UDA system. Over a period of 180 min, 80 percent of the encapsulated calcein was released from the UDA:UDG system (see Fig. 5, panel a, blue trace), whereas only 22 percent of the encapsulated calcein was released in case of the pure UDA membrane system (see Fig. 5, panel a, purple trace). This corroborates with what has been seen in related literature wherein Mansy S. *et al*. showed that addition of glycerol monoester of myristoleic acid (monomyristolein) to myristoleic acid (C14:1) system in 2:1 ratio, did increase the permeability of the membrane system^25^. Interestingly, the UDA:UDOH binary system was found to be impermeable to calcein, where none of the encapsulated calcein was released even after a period of 180 min (Fig. 5 panel a, red trace). The tertiary system of UDA:UDG:UDOH was found to possess moderate permeability to calcein, which was less permeable than the binary UDA:UDG system and the pure UDA system, but more permeable than the UDA:UDOH binary system, releasing only 14 percent of encapsulated calcein over 180 mins (see Fig. 5, panel a, green trace). While presence of UDG in the membrane led to an increase in the permeability, the presence of UDOH decreased the permeability of the UDA system. Furthermore, the encapsulation efficiency of all four C11 based membrane systems was estimated by encapsulating calcein. 0.5 mM calcein (below its self-quenching concentration) was encapsulated in all the four membrane systems and encapsulation efficiency was calculated as described in the Supplementary method section. The encapsulation efficiency of the tertiary mixed system was found to be the highest (around 10 percent) among all the four systems that were studied (Supplementary Fig. S16).

### Potential mechanism by which fatty alcohol and glycerol monoester confer stability on fatty acid membranes in the presence of Mg^2+^ ions

In order to understand the mechanism behind the increased stability of fatty acid membranes towards Mg^2+^ ions in the presence of the derivatives, the charge density on the membrane and the retention of the fatty acid moieties in the membranes was investigated. Fatty acid molecules stay in dynamic equilibrium and can therefore readily exchange between the membrane phase and the free monomers present in the solution^28^. The free negatively charged monomers can interact with Mg^2+^ ions and form crystalline aggregates, thus more amount of free fatty acids will lead to greater aggregation. Upon quantifying free fatty acid (see Experimental Section and Supplementary for more details), it was observed that the UDA system possessed the highest amount of free fatty acids (see Fig. 5 panel b, purple bar). The presence of UDG and UDOH showed a decrease in the dissociation of free UDA molecules into the solution, thereby stabilizing the mixed membranes (Fig. 5 panel b, blue and red bar). In case of OA based membrane systems, it was observed that both, OOH and GMO decrease the dissociation of OA into the solution, similar to what was observed in the UDA based systems (Supplementary Fig. S17). The free Mg^2+^ ions present in the solution also tend to interact with the negatively charged fatty acid vesicles and initiate the collapsing of vesicles. Therefore, a decrease in the net negative charge on the vesicle surface would lead to a weakening in the interaction with the Mg^2+^ ions.

The zeta potential measurements (see Method Section and Supplementary for details) revealed that the addition of UDG to the UDA membrane, decreased the net negative charge density significantly (Fig. 5 panel c, blue bar). The net negative charge density of the UDA:UDOH binary system was equal to that of the only UDA system (see Fig. 5 panel c, purple and red bar). The negative charge density on the tertiary UDA:UDG:UDOH system was found to be somewhere in between these, i.e higher than the UDA:UDG system but lower than the UDA and UDA:UDOH system (see Fig. 5 panel c, green bar). The change in the negative charge density can be explained by considering the size of the different head groups. Because of the bulky head group of the UDG moiety, less number of deprotonated fatty acid molecules would be present in a given unit surface area as opposed to what could potentially happen in the presence of UDOH. This observation is further strengthened when taking into account the permeability of the membrane systems as shown in Fig. 5 panel a. Similar pattern of zeta potential values was also observed for the oleic acid based membranes (Supplementary Fig. S17). Therefore, we found that both of the components, i.e. retention of fatty acid molecules in the membrane, and the change in the surface charge density, would contribute towards the increased stability of the mixed membrane systems in the presence of Mg^2+^ ions. Furthermore, the long chain alcohols can stabilize the fatty acid membranes by retaining the fatty acid molecules in the membrane, whereas the glycerol monoester not only increases the fatty acid retention in membrane, but also decreases the overall negative charge density.

### Multiple selection pressures (MSPs) and survivability of model protocell membranes as a function of their composition

**Figure 6:**
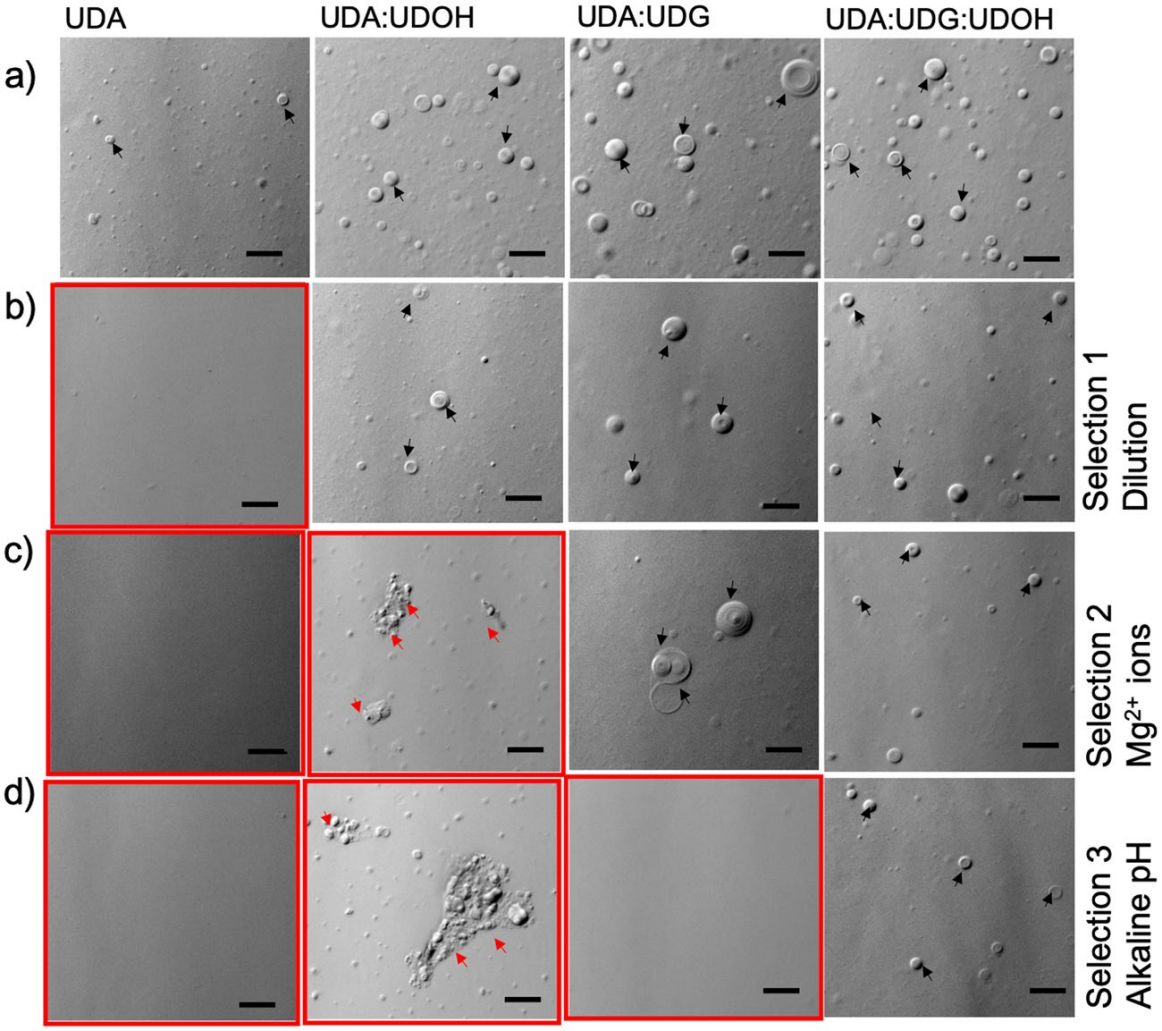
Membrane stability under Multiple selection pressures (MSPs), when applied consecutively. Panels b to d represent different prebiotically relevant selection conditions. (**a**) All four C11 based systems at a concentration of 60 mM at pH 8. (**b**) Dilution regime selection: All four C11 based systems diluted to a concentration of 20 mM at pH 8. (**c**) Stability in presence of Mg^2+^ ions: All four C11 based systems at 20 mM total lipid concentration at pH 8, in the presence of 14 mM Mg^2+^. (**d**) Stability at alkaline pH: All systems at 20 mM total lipid concentration in the presence of 14 mM Mg^2+^ and at an alkaline pH of 10. The red enclosures indicate absence of vesicles. The black and red arrows indicate vesicles and aggregates (crystalline aggregates and collapsed vesicles) respectively. The scale bar = 10 microns.

The MSP experiment (see method section and Supplementary information) revealed that even though the binary mixed systems (UDA:UDOH and UDA:UDG) could be more stable under a given selection pressure, when all the three selection pressures were applied sequentially, the tertiary system (UDA:UDG:UDOH) was the one that stood the best chance at survival. As shown in Fig. 6 panel a, all four C11 based systems formed vesicles at a lipid concentration of 60 mM at pH 8. When all the four systems were diluted to a final lipid concentration of 20 mM using a buffer of pH 8, the UDA system failed to form vesicles because of its high CVC requirement (near 35 mM), while the other three mixed systems continued to form vesicles (panel b). Next, out of the three mixed systems, only Mg-soap crystals and collapsed vesicles were observed in UDA:UDOH system when Mg^2+^ ions were added to the solution at a concentration of 14 mM (panel c). Among the other two mixed systems, UDA:UDG and UDA:UDG:UDOH, being less sensitive to Mg^2+^ ion concentration, continued to form vesicles. Finally, when the pH of the solution was adjusted to 10, the UDA:UDG system failed to assemble into vesicles. Consequently, only the tertiary system of UDA:UDG:UDOH was able to assemble into vesicles when all the selection pressures were applied back to back (panel d). Significantly, this observation was independent of the sequence of the selection pressures applied (Supplementary Figs. S18 to S22).

## Discussion

It is reasonable to assume that the environments of the early Earth would have been complex and replete with different kinds of amphiphiles that could readily assemble into membrane structures under pertinent conditions. The physico-chemical properties of these primitive compartments would have been largely affected by their environmental conditions. When the fitness of the different membrane systems was tested using dilution as the selection regime, it was observed that the incorporation of both of the derivatives, i.e. fatty alcohol and glycerol monoester moieties, decreased the CVC of the system. The UDA:UDG and the UDA:UDG:UDOH mixed systems were found to possess the lowest CVC among the four UDA based systems (Table 1). While, in the OA based systems, the OA:GMO system was found to have the lowest CVC which suggests that the influence of the glycerol monoester on lowering the CVC is possibly greater than the fatty alcohol moiety. A crucial aspect to highlight here is that three different methods were employed in our study to estimate the CVC of the systems. This is because use of any of the individual techniques in isolation is not sufficient to gain an understanding of the complete picture. Thus, three techniques were combined to confidently narrow down the CVC of the systems to a precise range.

As for the stability of the various systems in alkaline pH regimes, pure fatty acid systems were found to be extremely sensitive (see Fig. 1, panel a). The UDA:UDG system failed to form vesicles above a pH of  9. Whereas, the UDA:UDOH binary system and the UDA:UDG:UDOH tertiary system continued to assemble into vesicles even at pH 11 (Fig. 1). In case of the OA based systems, all three mixed systems (OA:GMO, OA:OOH, OA:GMO:OOH) were found to form vesicles even at pH 11 (Supplementary Fig. S4). These observations can be explained by factoring in the protonation status of the different species. As the pH increases, the fatty acid species get deprotonated, allowing it to hydrogen bond with the hydroxyl group of the alcohol or the glycerol head group, resulting in membrane assembly^3,6^. Hence, in the case of UDA:UDG:UDOH tertiary system, the membrane stability predominantly seems to stem from the UDOH moiety above pH 9 as the UDA:UDG binary system fails to assemble into vesicles above pH 9. In recently published work, Jordan S. F. *et al*. demonstrated that a mixture of fatty acid and 1-alkanol of C10 to C15 chain length can assemble into vesicles even at elevated temperature (~ 70 °C) in alkaline conditions (pH 6.5 to 12)^29^. As for the OA:OOH system, it does not form vesicles at pH 8, while the pure OA system can (Supplementary Fig. S4). This might potentially stem from the predominance of the protonated species at this pH, which hampers efficient hydrogen bonding.

When the stability of the membrane systems in question was evaluated against Mg^2+^ ion concentration, the UDA:UDG binary system was found to be the most resilient among the UDA based systems, followed by the UDA:UDG:UDOH (Supplementary Table S1). The UDA:UDOH was found to be the second most sensitive one after the pure UDA system. In the OA based systems, the OA:OOH and the OA:GMO:OOH systems were found to be equally stable towards higher Mg^2+^ ion concentrations. The OA:GMO binary system was found to be the second most sensitive one, after the only OA-based system (Supplementary Table S1). The greater stablizing effect of OOH, compared to that of GMO, could potentially be attributed to an increase in the chain length. As the chain length increases, parameters like membrane thickness, membrane packing, CVC etc. also change^3^. Previous studies have demonstrated that the presence of chelating agents such as citrate could stabilize oleic acid membranes in the presence of high concentrations (50 mM) of Mg^2+^ ion, while allowing for the function of the encapsulated ribozyme^30,31^. However, presence of chelated Mg^2+^ decreases the rate of the reaction significantly^30,32^. This becomes very relevant when low amount of Mg^2+^ ions are present in the environment, highlighting the disadvantage of chelated Mg^2+^ ions^32^. Meeting such high concentration of certain chelating agents, might have been difficult in the dilute regimes of the prebiotic Earth. It has been reported that, low concentration of free Mg^2+^ ions (4 to 15 mM) can facilitate ribozyme function^19,24,33^, which are compatible with the mixed membrane systems described in this study. Therefore, it seems reasonable to hypothesize that an increase in the membrane compositional heterogeneity, along with some chelation, could have provided a respite from the Mg^2+^ ion conundrum.

Compartments possessing very high or very low permeability would not have been suitable to support protocellular life forms. Rather, compartments possessing moderate permeability, which would have allowed the permeability of small molecules (like nucleotide monomer, dimer and amino acids etc), to the interior, without letting the internal components to permeate out, would have been more ideal. Results from our permeability experiments (Fig. 5 panel a) show that the incorporation of UDG into UDA membranes increases the membrane permeability when compared to the pure UDA membrane system. On the other hand, the addition of UDOH decreases the permeability of the system. Therefore, an optimally permeable membrane would have required a mixture of both of these derivatives, as observed in the tertiary system of UDA:UDG:UDOH. Because of the bulky head group, the glycerol monoester is thought to hinder efficient membrane packing, while also stabilizing membrane curvatures, which might have resulted from membrane solute interaction^25^. Thus, the permeability of the system increases. UDOH on the other hand, possess a small head group, which can potentially increase membrane packing, resulting in a decrease in the permeability. Basically, tuning the concentration of the various components could, in turn, enable the tuning of the permeability of a given model protocellular system that would have determined its suitability for a specific set of environmental parameters.

Interestingly, the C11 based tertiary system was found to possess the highest encapsulation efficiency compared to pure UDA, binary UDA:UDG and UDA:UDOH membrane systems (Supplementary Fig. S16). UDA also contains a terminal unsaturation, which might potentially facilitate the oxidative degradation of UDA. However, such oxidation reaction are known to occur at elevated temperature^34^. In order to check whether the UDA molecules degrade under our reaction conditions and time scales, the oxidation stability of UDA was also investigated for 12 hours at room temperature. Microscopy and TLC analyses, and quantification of the amount of bilayer present (using DPH) was carried to have a comprehensive idea about its stability towards oxidation (Supplementary method section). No degradation of UDA was observed in the aforesaid reaction conditions even after 12 hours of incubation (Supplementary Fig. S23).

In conclusion, our study highlights that the stability of model protocellular membranes is not necessarily a linear property of its compositional heterogeneity. This is because, it is governed differently by different prebiotically relevant parameters. Importantly, the effect of each head group on the stability of the membrane seems to depend on the environment and the selection pressure that the system is being subjected to. For example, at alkaline pH of 10 or above, the UDOH stabilizes UDA membranes, while UDG cannot. Whereas, in the presence of Mg^2+^ ions, the UDG stabilizes the UDA membranes more than the UDOH moiety. Overall, the tertiary system was found to be more stable in the presence of all the three selection conditions, especially when they were applied sequentially. When put together, these results indicate that different prebiotically pertinent selection pressures would have shaped the evolution of protocellular membranes in a specific manner that was predominantly determined by their composition. Significantly, when multiple selection conditions act upon concurrently, the tertiary systems, being more complex, possess a better chance at survival, highlighting that complex mixed membrane systems would have been necessary to support the emergence of protocellular life forms on the early Earth.

## Methods

Experimental methods are described in more detail in the Supplementary Information section.

### Vesicle solution preparation

The vesicle solutions were prepared by dissolving the desired amount of the fatty acid and its derivatives in chloroform at a concentration of 10 mg/ml. The chloroform solution was dried under nitrogen gas flow to prepare a dry lipid film. It was then kept under vacuum for five to six hours to make sure that no trace amount of chloroform remained. Subsequently, different buffers (bicine or CHES) of desired pH were used to rehydrate the thin film to form the vesicles. This vesicle suspension was heated for one hour at 60 °C to maximize vesicle formation.

### Microscopic analysis

Lipid samples were observed under 20X and 40X magnification using a Differential Interference Contrast (DIC) microscope AxioImager Z1 (Carl Zeiss, Germany), (NA = 0.75) to observe the presence of different assemblies (vesicles, oil droplets, crystalline aggregates etc.). Typically, 10 μL of lipid solution was spread on a glass slide, followed by placing an 18 × 18 mm coverslip on top of it and covering the four sides with liquid paraffin to decrease the motion of the lipid solution. Thereafter, the slide was immediately observed under the microscope.

### CVC estimation

In order to determine the CVC of the membrane systems, 1,6-diphenyl-1,3,5-hexatriene (DPH) was used as a membrane probe. DPH fluorescence was plotted as a function of lipid concentration. The concentration of lipid where a sudden increase in fluorescence is observed (the inflection point) represents the CVC of the system^35^. Additionally, turbidity assay^7^ and microscopy were also performed to confirm the CVC results (see the Supplementary for more details).

### Stability in alkaline pH regimes

Effect of compositional heterogeneity on the formation and stability of the protocell membranes was investigated from pH 7 to 11. Optical microscopy was used to discern the nature of the higher order assemblies as a function of pH (see the Supplementary for more details).

### Stability against Mg^2+^ ion

Optical microscopy and dynamic light scattering (DLS) spectroscopy were used to check the vesicle stability. The concentration of Mg^2+^ ion at which the average size of the vesicle population increases, in comparison to the initial size (when no Mg^2+^ is added), was considered to be the aggregation-inducing concentration (Mg^2+^_AIC_)^36^. Additionally, optical microscopy was also used to confirm the presence of different structures such as crystalline aggregates (Mg-soap) and collapsed vesicles (see the Supplementary for more details).

### Permeability assay

In order to check the permeability of model protocell membranes, calcein leakage assay was carried out. In a typical experiment, calcein was encapsulated in the different vesicular systems above its self-quenching concentration (30 mM), and its release was measured over time to estimate permeability of the membrane systems, which is in turn a reflection of its dynamicity^36^ (see the Supplementary for more details).

### Free fatty acid quantification

The amount of free fatty acid monomers present in the solution was estimated for all the eight membrane systems using liquid chromatography, coupled with mass spectrometry. The amount of free fatty acid for each of the heterogeneous systems was normalised to their respective pure fatty acid system (see the Supplementary for more details).

### Membrane charge estimation

The surface charge density of the membranes was estimated by measuring their zeta potential (ζ) (see the Supplementary for more details).

### Multiple selection pressure (MSP)

The stability of different membrane systems under MSP was investigated using C11 based systems. Three selection pressures including dilution regime, formation of vesicles in alkaline pH and stability in the presence of Mg^2+^ ions, were applied to the membranes in question in a sequential manner. Furthermore, to understand if there was any influence coming from the order of the applied selection pressures, a total of six different combinations of the aforesaid selection pressures were applied by varying their sequence. After applying each selection pressure, the solution was observed under microscope to check for the presence of vesicles (see the Supplementary for more details).

## Supplementary information

Supplementary Information.
