# Supplementary material for: Compositional heterogeneity confers selective advantage to model protocellular membranes during the origins of cellular life
Source: Sci Rep. 2020 Mar 11;10:4483. doi: 10.1038/s41598-020-61372-w (PMC7066133; doi:10.1038/s41598-020-61372-w)
Supplement: Supplementary file 1 — Supplementary Information. [file 41598_2020_61372_MOESM1_ESM.pdf]

Supplementary Information

Compositional heterogeneity confers selective advantage to model protocellular membranes during the origins of cellular life

Susovan Sarkar‡, Shikha Dagar‡, Ajay Verma‡, Sudha Rajamani‡*

‡Department of Biology, Indian Institute of Science Education and Research, Pune 411008, India

*Correspondence: srajamani@iiserpune.ac.in; Tel.: +91-20-2590-8061

Table of Contents

Materials...4

Methods

CVC Estimation……………………………………………………………………......5

Membrane stability in alkaline pH regimes…………………………………….....…6

Membrane stability in presence of Mg^{2+} ions…………………………………...…7

Rescue of vesicles from Mg^{2+} ion induced aggregates…………………………………8

Permeability Assay………………………………………………………………........…8

Encapsulation efficiency estimation……………………………………………......10

Zeta potential measurement…………………………………………………………...11

LC-MS analysis of free fatty acid…………………………………………………......…12

Multiple Selection Pressures (MSPs)……………………………………………......13

Oxidation stability of UDA (C11)..…………………………………………………........14

Table S1 (membrane stability in presence of Mg^{2+} ions)……...................................16

Figures S1 (structures of amphiphiles)……………………………………………......17

Figures S2 (Stability in alkaline pH regime, all systems)……………………………...18

Figures S3 (Stability in alkaline pH regime, C11 based system)……………………..19

Figures S4 (Stability in alkaline pH regime, C18 based system)……………………..20

Figures S5 (Stability in alkaline pH regime, C18 based system)……………………..21

Figures S6 (Absorbance of C11 and C18 based system)……………………….…....22

Figures S7 (CVC estimation of C11 based system using DPH)……………………...23

Figures S8 (CVC estimation of C18 based system using DPH)………………………24

Figures S9 (CVC estimation of C11 based system by measuring Absorbance)……………………………………………………………………………..25

Figures S10 (CVC estimation of C18 based system by measuring Absorbance)……………………………………………………………………………..26

Figures S11 (CVC estimation of C11 and C18 based system using microscopy)….27

Figures S12 (Mg^{2+}_{AIC} estimation of C18 based system using DLS)……………….....28

Figures S13 (Mg^{2+}_{AIC} estimation of C18 based system using microscopy) …………29

Figures S14 (Rescue of vesicles form Mg^{2+}-induced aggregates using EDTA) 30

Figures S15 (Size exclusion chromatography and calcein encapsulation)…………32

Figures S16 (Encapsulation efficiency estimation)……………………………..…...33

Figures S17 (LC-MS analysis and Zeta potential estimation)……………………..…34

Figures S18 (membrane stability under multiple selection pressure)…………..…..35

Figures S19 (membrane stability under multiple selection pressure)………………..36

Figures S20 (membrane stability under multiple selection pressure)………………..37

Figures S21 (membrane stability under multiple selection pressure)………………..38

Figures S22 (membrane stability under multiple selection pressure)………………..39

Figures S23 (UDA oxidation stability)……………………………………………......40

Materials

Magnesium chloride hexahydrate ($MgCl_2 \cdot 6\ H_2O$, 203.30 g/mol), sodium hydroxide (NaOH, 39.997 g/mol), hydrochloric acid (HCl, 37 %, 36.46 g/mol), bicine ($C_6H_{13}NO_4$, 163.17 g/mol), CHES ($C_8H_{17}NO_3S$, 207.287 g/mol), calcein (622.55 g/mol) and triton X100 (647 g/mol) were purchased from Sigma Aldrich (Bangalore, India) and used without further purification. All the fatty acids mentioned in this study, namely oleic acid (cis-9, $C_{18}H_{34}O_2$, 282.47 g/mol), oleyl alcohol (cis-9, $C_{18}H_{36}O$, 268.478 g/mol), glycerol 1-monooleate (cis-9, $C_{11}H_{40}O_4$ 356.547 g/mol), undecylenic acid ($C_{11}H_{20}O_2$, 184.279 g/mol), undecylenyl alcohol ($C_{11}H_{22}O_1$**,** 170.29), glyceryl 1-undecylenate ($C_{14}H_{26}O_4$**,** 258.35 g/mol) and myristoleic acid (226.36 g/mol) were purchased from Nu-Chek-Prep (Elysian, MN, USA) and used without further purification. All other chemicals were purchased from Sigma Aldrich (Bangalore, India) and used without further purification. All the experiments were carried out using Nanopure (18 MΩ-cm) water.

Methods

Estimation of critical vesicular concentrations (CVCs)

Three different methods were used to estimate the CVC of the membrane systems in question. The first method involved using 1,6-diphenyl-1,3,5-hexatriene (DPH), a hydrophobic fluorescent dye, that can partition into the hydrophobic region of the membrane. Upon partitioning, its fluorescence increases several folds. The increase in fluorescence is directly proportional to the lipid concentration. DPH fluorescence also depends on the kind of lipid used, amount of dissolved ions, and incubation time. The lipid suspension used in the experiment was prepared by diluting a stock lipid suspension with a pH appropriate buffer. For the C11 and C18 based systems, 200 mM bicine buffer of pH 8, and 100 mM CHES buffer of pH 9, respectively, was used to rehydrate the dried lipid film and to prepare further dilutions. The lipid suspension was then sonicated to form a homogeneous mixture of small unilamellar vesicles. In a typical reaction, 1.8 µL of 400 µM methanol solution of DPH, was added into 180 µL of C11 based lipid suspension to achieve 4 µM final concentration of DPH in the solution. For all the C18 based systems the final DPH concentration was decreased to 2 µM to account for reduced lipid concentration (long chain fatty acids tend to have much lower CVCs than small chain fatty acids). The lipid suspensions were prepared by diluting the stock lipid suspension with appropriate buffer. After adding the DPH into the lipid suspension, the mixture was kept at 40° C at a constant rotation of 700 rpm for 30 minutes to increase the partitioning of DPH in to the membrane. Post-incubation, the suspension was transferred to a 96-well plate. The fluorescence was measured using a 96-well plate reader on Thermo Scientific Varioskan Flash multimode reader (Thermo Scientific, Singapore) by exciting the samples at 350 nm and measuring the emitted light at 452 nm. To corroborate the

CVC values obtained from this fluorescence assay, the absorbance (at 400 nm) of the lipid suspension was also measured at 400 nm, which is a widely used technique in the field, for reporting CVCs. The lipid concentration at which the turbidity of the system increases sharply is considered to be an indication of formation of vesicles, and hence is considered as the CVC of the system. A UV-1800 UV-Vis Spectrophotometer (Shimadzu Scientific Instruments Inc., Columbia, USA) was used to check the absorbance of the lipid suspensions, which was indicative of higher order structure formation. The presence of vesicles was further confirmed by microscopy at 40X magnification, as described in the aforementioned section.

Evaluation of formation of vesicles in alkaline pH regimes

The ability of different lipid systems to assemble into vesicles was evaluated from pH 7 to 11, at intervals of 0.5 pH units (e.g. pH 7, 7.5, 8 and so on). 6 and 60 mM lipid concentration was used for the C18 and C11 based systems, respectively. Typically, the dried lipid films were hydrated with a buffer of appropriate pH so as to cover the whole pH range mentioned. This was done considering the fact that different buffers have their own range of buffering capacity. For example, 200 mM bicine was used to prepare buffers in the pH 7 to 9 regime, while 200 mM CHES was used for pH 9.5 to 11 regime. The scattering of the suspension at 400 nm was used as a proxy to gauge the presence of vesicles and oil droplets that were present in the suspension using UV-1800 UV-Vis Spectrophotometer (Shimadzu Scientific Instruments Inc., Columbia, USA). The same samples were subsequently observed under microscope at 40X magnification to discern the nature of the higher order assemblies (e.g. vesicles, droplets etc).

Stability of vesicles in the presence of Mg^{2+} ions

In order to check for the stability of the vesicles in the presence of Mg^{2+} ion, Dynamic Light Scattering (DLS) was used. In a typical experiment, the vesicle suspension was extruded 15 times through a 200 nm size cut-off polycarbonate membrane using Avanti mini extruder (Avanti Polar Lipids Inc., Alabaster, AL, USA). The Mg^{2+} ions were then added to the lipid suspension by adding a desired volume of $MgCl_2$ stock solution, prepared in the respective buffer. 100 mM CHES buffer of pH 9 and 200 mM bicine buffer of pH 8 were used to prepare the C18 and C11 based vesicle suspensions, respectively. The suspension was then set aside for 15 min to equilibrate, after which the average size of the particles in the suspension was measured using Zetasizer Nano ZS90, (Malvern Panalytical Ltd., Malvern, UK). The average size of the population was plotted against the Mg^{2+} ion concentration, so as to estimate Mg^{2+} ion induced fatty acid aggregation. The total lipid concentration was kept at 2 mM for the four C18 based systems. 20 mM lipid suspension was used for all the three heterogeneous C11 based systems to prevent concentration induced vesicle aggregation. However, for the homogenous UDA system, the lipid concentration was kept at 60 mM due to its intrinsically high CVC. As for the microscopy analysis, the lipid concentration was kept the same as was used for the DLS experiment. Desired amount of Mg^{2+} ion concentration was obtained by adding different volumes of the $MgCl_2$ stock solution in the lipid suspension. Presence of different forms of aggregates, namely, crystalline aggregates and collapsed vesicles, were checked for using DIC microscopy.

Reformation of vesicles from Mg^{2+} ion induced aggregates in the presence EDTA

In order to check to check if the lipid aggregates, formed in presence of Mg^{2+} ions (magnesium soap crystals, collapsed vesicles) can be rescued to form vesicle again, EDTA (Ethylenediaminetetraacetic acid), was added to the suspension. C11 based membrane system was used for this study. 60 mM lipid suspension was used for all the four C11 based membrane systems. 200 mM bicine buffer of pH 8 was used to prepare all vesicle suspensions. Desired amount of Mg^{2+} ion concentration was obtained by adding different volumes of the $MgCl_2$ stock solution in the lipid suspension and was observed under the microscope to check for Mg^{2+} ion induced aggregates (magnesium soap crystals, collapsed vesicles). After that EDTA solution was added in the suspension to reach 1:1 and 1:2 of Mg^{2+} ion to EDTA molar ratio concentration. The suspension was then mixed and kept to equilibrate. Thereafter the suspension was observed under the microscope.

Permeability Assay

In order to determine the permeability of the different C11 based membrane systems, calcein leakage assay was used. Calcein is a small polar molecule with an excitation and emission wave length of 495 and 515 nm, respectively, and it gets self-quenched at a high concentration. This property was used to carry out this study by encapsulating calcein above its self-quenching concentration. This was done by rehydrating the dried lipid film with 200 mM bicine buffer pH 8, containing 35 mM of calcein. For all the three heterogeneous C11 based systems, 90 mM of total lipid concentration was used. However for the homogenous UDA system, the lipid

concentration was kept at 150 mM due to its relatively high CVC. Interestingly, the pH of the suspension dropped after dissolving the dried fatty acid film in the aforementioned mix of buffer and calcein. Therefore, the pH of the suspension was readjusted by adding NaOH solution. The suspension then went through four freeze-thaw cycles to increase the encapsulation efficiency.

To confirm the encapsulation of calcein in the vesicles, the crude suspension was observed under microscope, using both, fluorescence and DIC, as shown in Figure S11 B. Thereafter, the calcein encapsulated vesicle suspension was extruded 15 times through a 200 nm size cut-off polycarbonate membrane using Avanti mini extruder (Avanti Polar Lipids Inc., Alabaster, AL, USA) and was loaded on to a size exclusion column (20 cm X 1 cm) packed with Sephadex G-50 fine beads. The column was pre-equilibrated with the mobile phase, which is 200 mM bicine buffer containing just empty lipid vesicles. For each lipid system, the mobile phase contained the same lipid composition and ratio, but at a slightly higher concentration than their CVC, to prevent the lysis of the calcein encapsulated vesicles in the column. Fractions were then collected manually and loaded on to a 96-microwell plate (about 220 µL/well). The fluorescence was measured using a 96-well plate Varioskan Flash multimode reader (Thermo Scientific, Singapore), by exciting the samples at 495 nm and measuring the emitted light at 515 nm. The vesicles with encapsulated calcein eluted in the early fractions (fraction number 11 to 16), and the unencapsulated calcein eluted in the later fractions (fraction number 43 to 57), as shown in Figure S11 a. The fluorescence was monitored continuously for three hours. After that, 2 µL of Triton 100X was added in each well to rupture the vesicles and release the remaining encapsulated calcein, which led to maximum fluorescence.

The percentage of encapsulation was calculated by using the following equation.

Encapsulation (%) = $100 * (1 - \frac{Ft - F0}{Ff - F0})$

Where, $F0$ is the fluorescence at time zero, Ft is the fluorescence at time t and Ff is the final fluorescence after the addition of Triton 100X.

Encapsulation efficiency estimation

In order to determine the encapsulation efficiency of the different C11 based membrane systems, calcein was used. Calcein was encapsulated in the vesicle by rehydrating the dried lipid film with 200 mM bicine buffer pH 8, containing 0.5 mM of calcein (below its self-quenching concentration). For all the three C11 based mixed membrane systems, 90 mM of total lipid concentration was used. However, for the pure UDA system, the lipid concentration was kept at 140 mM due to its relatively high CVC. The suspension was then undergone through three freeze-thaw cycles to increase the encapsulation efficiency. The encapsulated vesicle suspension was then extruded 15 times through a 200 nm size cut-off polycarbonate membrane using Avanti mini extruder (Avanti Polar Lipids Inc., Alabaster, AL, USA) and was loaded on to a size exclusion column (20 cm X 1 cm) packed with Sephadex G-50 fine beads. The column was pre-equilibrated with the mobile phase, which is 200 mM bicine buffer containing just empty lipid vesicles. For each lipid system, the mobile phase contained the same lipid composition and ratio, but at a slightly higher concentration than their CVC, to prevent the lysis of the calcein encapsulated vesicles in the column. Fractions were then collected manually and loaded on to a 96-microwell plate (about 200 μL/well). The vesicles with encapsulated calcein

eluted in the early fractions and the unencapsulated calcein eluted in the later fractions. After that, 2 μL of Triton 100X was added in each fraction to rupture the vesicles. The fluorescence of all the fractions were measured using a 96-well plate Varioskan Flash multimode reader (Thermo Scientific, Singapore), by exciting the samples at 495 nm and measuring the emitted light at 515 nm.

The encapsulation efficiency was calculated by using the following equation.

Encapsulation efficiency (%) = $100 * (\frac{Fv}{Fv+Ff})$

Where, Fv is the total fluorescence of all vesicle encapsulated fractions and Ff is the total fluorescence of all unencapsulated calcein fractions collected during the size-exclusion.

Zeta potential measurement of the lipid suspensions

To determine the negative charge density on the vesicles, the lipid suspension was extruded 15 times through a 200 nm size cut-off polycarbonate membrane using Avanti mini extruder (Avanti Polar Lipids Inc., Alabaster, AL, USA). For all the four C18 based systems, a total of 2 mM lipid suspension was used. In case of all the three mixed C11 based systems and homogenous UDA system, 20 mM and 60 mM lipid suspensions, respectively, were used. The lipid suspensions were kept for one hour to equilibrate before the actual measurement was taken. Post equilibration 600 ul of lipid suspension was loaded in a cuvette and the zeta potential readings were acquired using a Zetasizer Nano ZS90 (Malvern Panalytical Ltd., Malvern, UK).

LC-MS analysis of free fatty acid

Sample preparation

In case of C11 based systems, 60 mM and 90 mM lipid concentration was used, for the UDA and the three mixed systems, respectively. This was done to keep the concentration of the fatty acid i.e. UDA constant (60 mM) across all four systems. The lipid suspension was prepared in 200 mM bicine buffer of pH 8. Similarly, for the C18 based systems, 20 mM and 30 mM lipid concentration was used for the homogenous OA and the three mixed systems to keep the concentration of the oleic acid constant (20 mM) across all four systems. The lipid suspension were prepared in 100 mM CHES buffer of pH 9. 500 μL of each sample was loaded onto a Vivaspin 2 centrifugal concentrator, with a molecular weight cut-off of 3 kDda and centrifuged at 5,000g for 15 min. Typically, 50 μL of the filtrate was collected. This filtrate was then acidified by adding 5 μl of formic acid. The free fatty acids present in the filtrate was then extracted by adding 400 μL of 2:1 choloroform:methanol solution. This solution also contained myristoleic acid of 10 μM as an internal standard. The extraction was carried out by vortexing the solution rigorously and then spinning it at 3000g for 2 mins. The organic phase was withdrawn carefully and was dried under a stream of nitrogen gas, and subsequently re-dissolved into 400 ul of 2:1 choloroform:methanol. A fraction of this was loaded on to the column (details in the below section).

Fatty acid quantification

Separation of fatty acids was carried out using a Luna C18 column from Phenomenex, Torrance, CA, USA (dimensions: 4.6 X 250 mm, 5 nm particle size). The solvent system used for the liquid chromatography part was as follows: Buffer A

used was 95:5 of water : methanol (vol/vol) + 0.1% ammonium hydroxide, and Buffer B was 60:35:5 of isopropanol: methanol : water (vol/vol) + 0.1% ammonium hydroxide. A typical LC run's total time was 22 minutes long. The gradient involved an increase in solvent B concentration from 5% to 100% in 4 minutes, followed by an isocratic phase of 100% of solvent B for fifteen minutes. This was followed by an equilibration phase for four minutes with solvent A, all of which was done at a constant flow rate of 0.4 mL/min. The undecylenic acid and oleic acid (from the respective reaction mixtures), and the myristoleic acid internal standard, eluted at 15.66, 15.75 and 15.84 minutes, respectively. The mass spectrometry was carried out on a Sciex X500R QTOF mass spectrometer (MS) fitted with an Exion-LC series UHPLC (Sciex, CA, USA), using Information Dependent Acquisition (IDA) scanning method. All the mass acquisitions were performed using Electron spray ionization (ESI) in the negative mode with the following parameters: turbo spray ion source, medium collision gas, curtain gas = 30 L/min, ion spray voltage = -4500 V (negative mode), at 300 °C. TOF-MS acquisition was done at declustering potential of -80 V, while using -10 V collision energy. The acquired data was analyzed using the Sciex OS software (Sciex, CA, USA; University of Florida, FL, USA). The presence of a specific species was confirmed by the presence of precursor mass within 3 ppm error range. The fatty acid was quantified by taking the ratio of the area under the corresponding fatty acid peak, with respect to the area of internal standard peak.

Membrane stability under Multiple Selection Pressures (MSPs)

To evaluate the stability of membranes as a function of their composition under multiple selection pressures, C11 based membrane systems were used. Three

environmental selection pressures, i.e. A) vesicle formation in alkaline pH regime. B) vesicle stability in dilution regimes, and C) vesicle stability in the presence of Mg^{2+} ions, were applied to the systems in a sequential manner. In order to understand if there was any effect coming from the sequence of the applied selection on the fitness of the vesicles, the selection pressures were applied in all possible combinations. Therefore, a total of six different sequential combinations were investigated. All the four C11 based systems were prepared in 200 mM bicine buffer at pH 8 with the lipid concentration kept at 60 mM. Typically, 300 µL of each vesicle suspension was taken in a centrifuge tube. In one of the MSPs sequence combinations tested, first the lipid suspension was diluted with 200 mM bicine buffer of pH 8 in order to check the stability of the vesicles on dilution. In the next step, the pH was increased by adding desired volume of 3M NaOH solution. It was added to all the lipid suspensions to bring the final pH to 10. In the final step, the stability was checked in the presence of Mg^{2+} ions by adding desired amount of $MgCl_2$ solution to reach a concentration of 14 mM Mg^{2+}. After the application of each aforesaid selection pressure, the lipid suspension was observed under microscope at 40X magnification to check for the presence of vesicle and unordered aggregates (crystalline aggregates and collapsed vesicles). In the same manner, the other five MSP combinations were also tested and the membrane systems were evaluated using microscopic analysis.

Oxidation stability of UDA (C11)

In order to check for the oxidation stability of UDA, thin layer chromatography (TLC), DIC microscopy and bilayer quantification (using DPH fluorescence assay) was

carried out. The stability of UDA vesicle suspension was monitored for 12 hours at room temperature in aerobic (in presence of oxygen) and in anaerobic (in absence of oxygen) condition. UDA vesicle suspension was prepared by dehydrating the lipid dry film with 200 mM bicine buffer of pH 8. For the anaerobic vesicle suspension, degassed buffer was used and the rehydration was carried out inside an anaerobic chamber. For the aerobic vesicle suspension, rehydration was carried out in air. Both of the suspensions were heated at 50 °C and mixed well followed by incubating at room temperature. Samples were taken out and analyzed at the beginning and after 12 hours.

The vesicle suspension for both the conditions (anaerobic and aerobic) i.e. 0th and 12th hour was observed under the microscope to check for the presence of vesicles. The samples corresponding to 0th and 12th hour time points for both of the conditions, were also run on a TLC plate to look for the oxidation product of UDA, if any. 5:4:1 toluene:choloroform:methanol was used as the mobile phase and the normal phase silica plate was used as the stationary phase. Primuline dye was used to stain the TLC plate and it was observed under a UV light (365 nm) using a handheld UV lamp. Finally, DPH (350/452 nm) was used to quantify the amount of bilayer in the different time points for both of the conditions, to see if there in any significant decrease in the amount of bilayer because of oxidative degradation.

Table S1: Procellular membrane stability in presence of Mg^{2+} ions.

Summary of aggregation-inducing Mg^{2+} ion concentration (Mg^{2+}_{AIC}) for all different C18 and C11 based systems using two different assays: Columns 2 and 3 provide a comparison of the difference in the Mg^{2+}_{AIC} estimation using DLS and microscopy assay, respectively. The fatty acid to overall derivative molar ratio was kept to 2:1. UDA, undecylenic acid; UDG, glyceryl 1-undecylenate; UDOH, undecylenyl alcohol; OA, oleic acid; GMO, glycerol 1-monooleate; OOH, oleyl alcohol.

Mg^{2+} ion (in mM) induced aggregation formation		
System used	DLS analysis	Microscopy
UDA	3	3
UDA:UDOH	8	8
UDA:UDG	16	12
UDA:UDG:UDOH	14	12
OA	3.5	2
OA:OOH	6	6
OA:GMO	5	4.5
OA:GMO:OOH	6	6

Figures S1 to S17

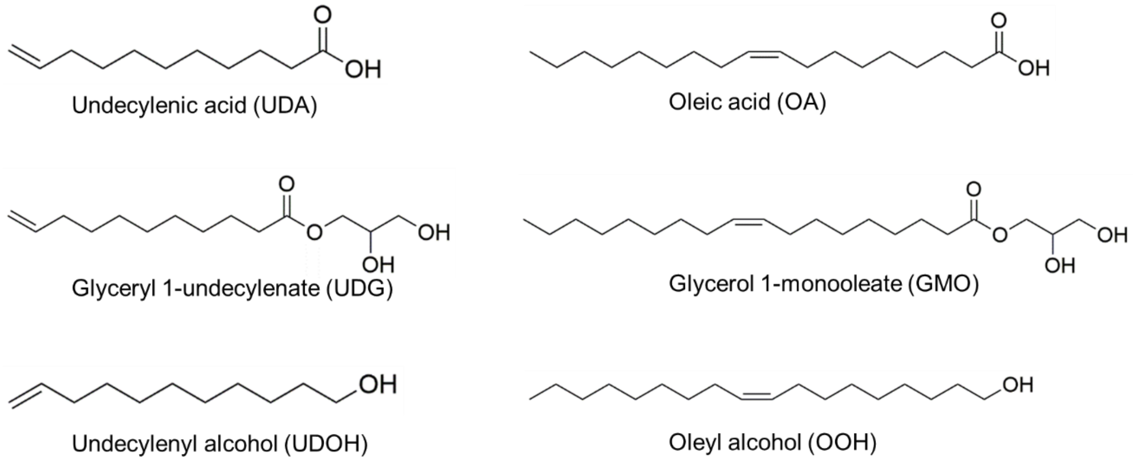

Figure S1: Structures of different amphiphiles, i.e. fatty acids (C11 and C18) and their derivatives used in the present study. All the structures were drawn using ChemDraw Professional.

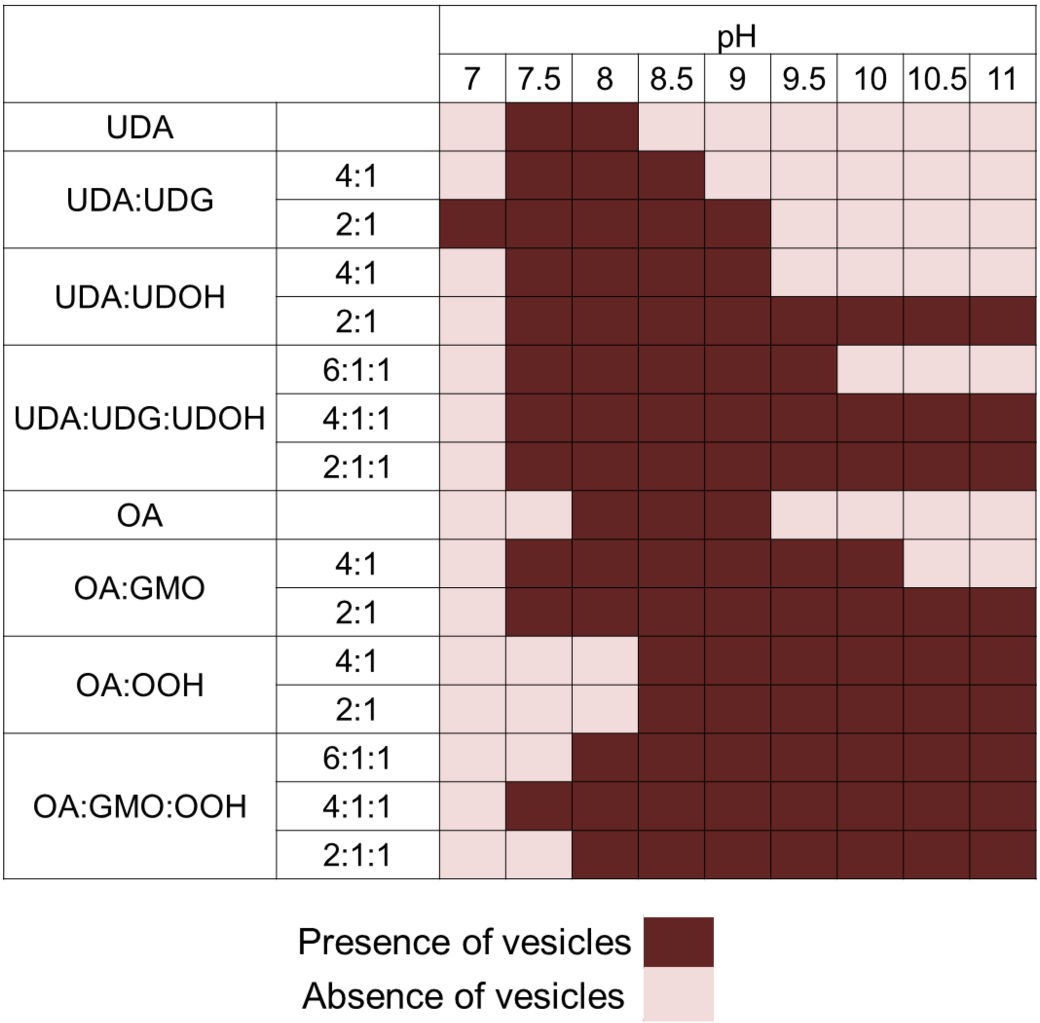

Figure S2: Formation of membrane under alkaline pH regimes

The ability of the different membrane systems to form vesicles over varying pH regimes has been illustrated in this figure. The ability of a system to form vesicles over a range of pH is represented in the matrix. n = 3. UDA, undecylenic acid; UDG, glyceryl 1-undecylenate; UDOH, undecylenyl alcohol; OA, oleic acid; GMO, glycerol 1-monooleate; OOH, oleyl alcohol.

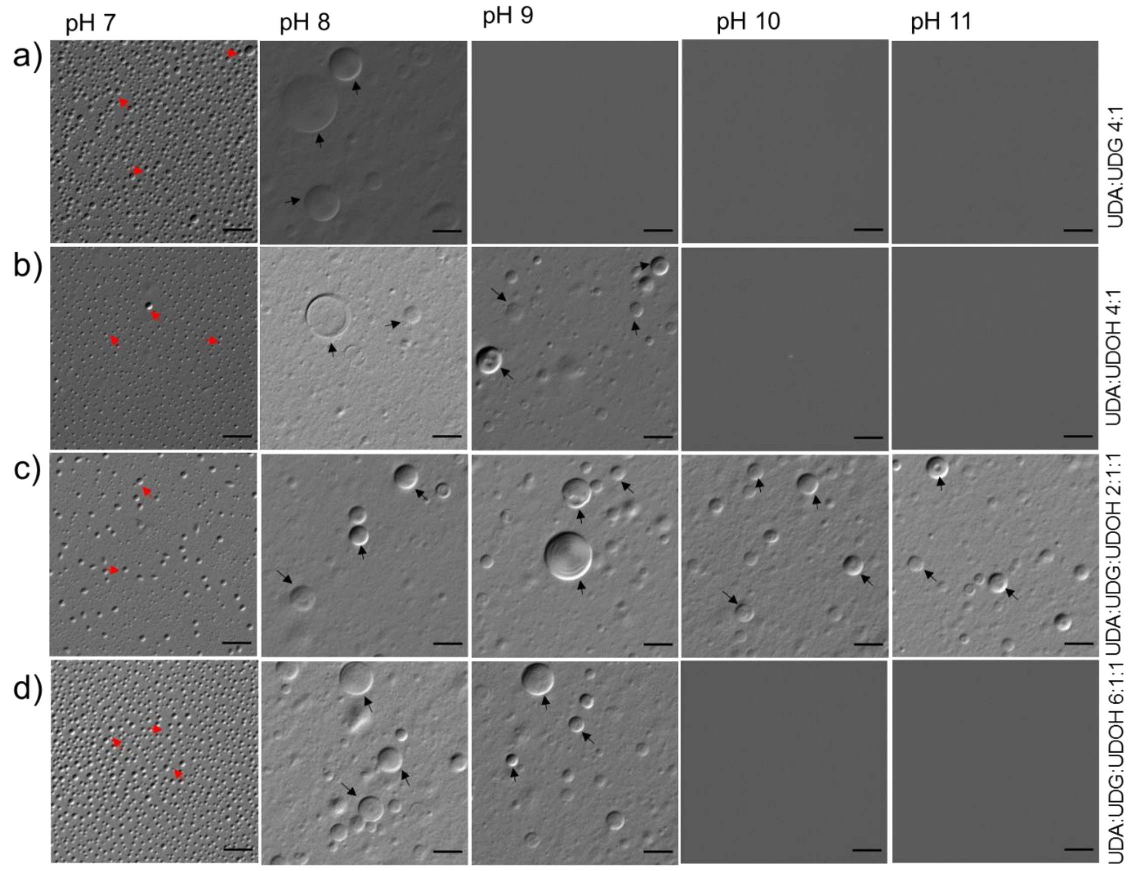

Figure S3: Formation of membrane under alkaline pH regimes

Microscopic analysis of C11 based membrane systems. These images demonstrate the formation of vesicles and oil droplets depending on the pH of the surrounding environment. Panels a to d show the four different C11-based systems, i.e, a) UDA and UDG mixed system in 4:1 ratio; b) UDA and UDOH mixed system in 4:1 ratio; c) UDA:UDG:UDOH mixed membrane system in 2:1:1 ratio and d) UDA:UDG:UDOH mixed membrane system in 2:1:1 ratio. The black and red arrows indicate vesicles and oil droplets, respectively. The scale bar in all the images is 10 microns. UDA, undecylenic acid; UDG, glyceryl 1-undecylenate; UDOH, undecylenyl alcohol.

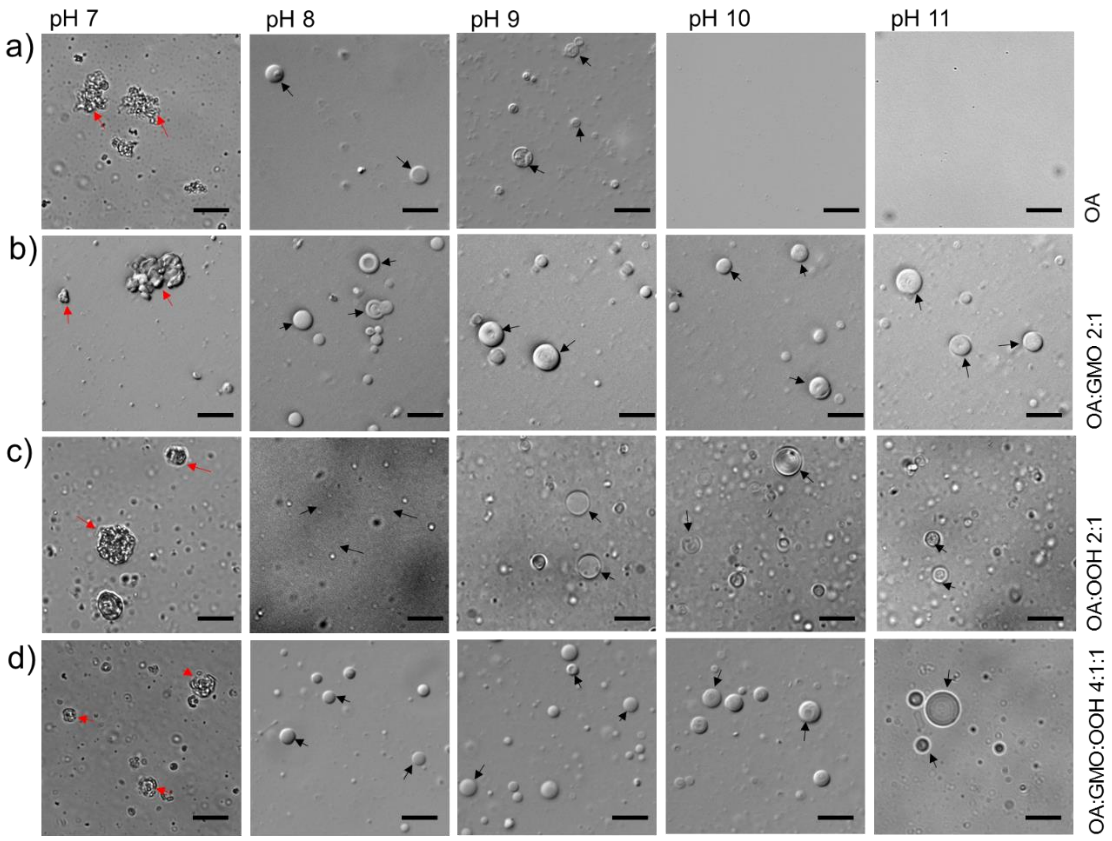

Figure S4: Formation of membrane under alkaline pH regimes.

Microscopic analysis of C18 based membrane systems. These images demonstrate the formation of vesicles and oil droplets depending on the pH of the surrounding environment. Panels a to d show the four different C18 based systems, i.e, a) pure OA system; b) OA and GMO mixed system in 2:1 ratio; c) OA and OOH mixed system in 2:1 ratio and d) OA:GMO:OOH mixed membrane system in 4:1:1 ratio. The black and red arrows indicate vesicles and oil droplets, respectively. The scale bar in all the images is 10 microns. OA, oleic acid; GMO, glycerol 1-monooleate; OOH, oleyl alcohol.

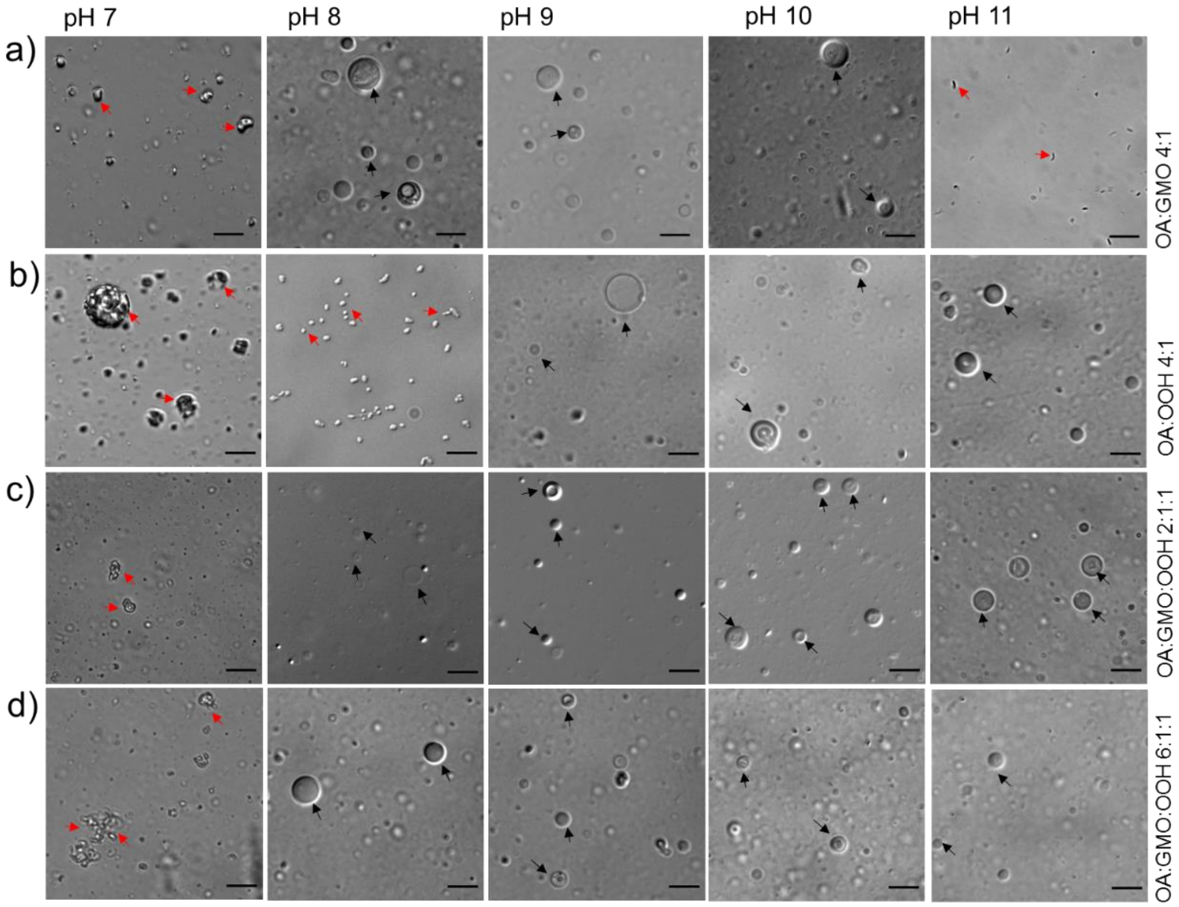

Figure S5: Formation of membrane under alkaline pH regimes.

Microscopic analysis of C18 based membrane systems. These images demonstrate the formation of vesicles and oil droplets depending on the pH of the surrounding environment. Panels a to d show the four different C18 based systems, i.e, a) OA and GMO mixed system in 4:1 ratio; b) OA and OOH mixed system in 4:1 ratio; c) OA:GMO:OOH mixed membrane system in 2:1:1 ratio and d) OA:GMO:OOH mixed membrane system in 6:1:1 ratio. The black and red arrows indicate vesicles and oil droplets, respectively. The scale bar in all the images is 10 microns. OA, oleic acid; GMO, glycerol 1-monooleate; OOH, oleyl alcohol.

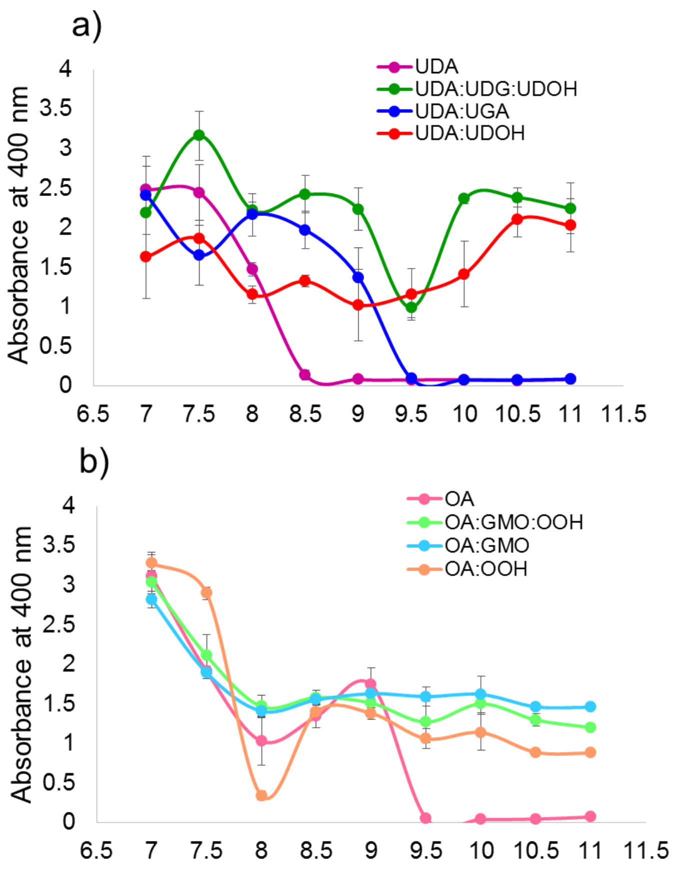

Figure S6: Measuring absorbance at 400 nm to check the formation of membrane under alkaline pH regimes.

The absorbance of the systems at 400 nm is plotted as a function of the pH. Panel a and b represent the different C11 and C18 based systems, respectively. Decrease in absorbance indicates the formation of micelles in the suspension, which cannot scatter light at 400 nm. The ratio of fatty acid to its respective glycerol monoester and/or alcohol was maintained at 2:1. n = 3; error bars represent standard deviation (s.d.). UDA, undecylenic acid; UDG, glyceryl 1-undecylenate; UDOH, undecylenyl alcohol; OA, oleic acid; GMO, glycerol 1-monooleate; OOH, oleyl alcohol.

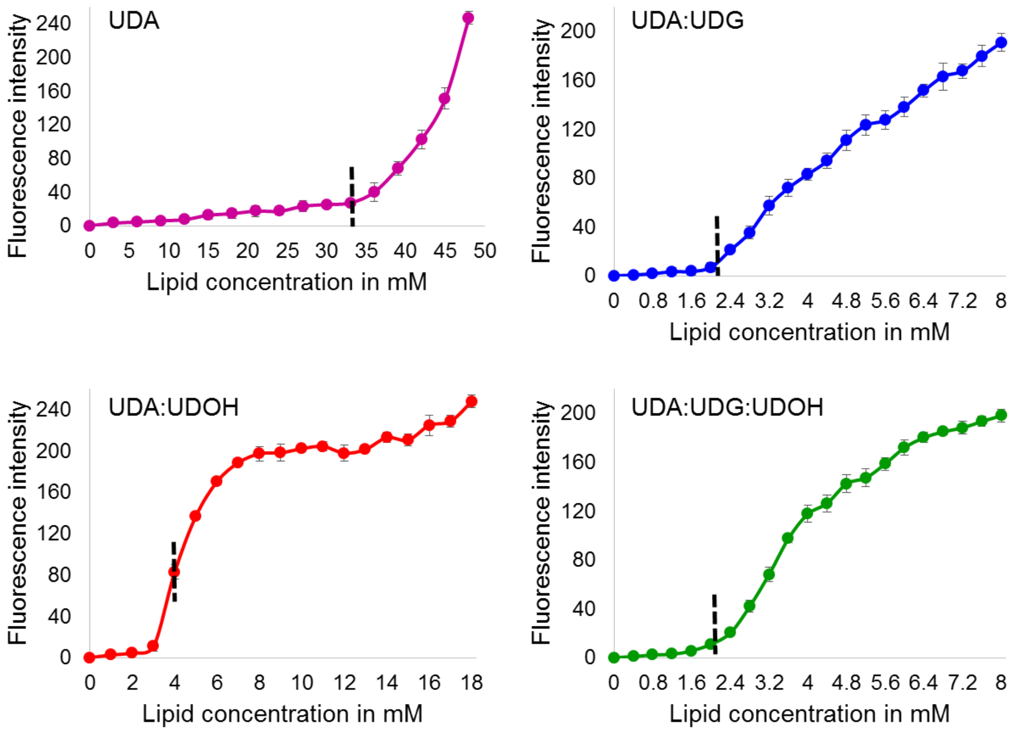

Figure S7: CVC estimation of different C11 based systems by fluorescence assay.

The increase in fluorescence is plotted as a function of total lipid concentration (fatty acid and derivatives). The inflection point, which is a read out of the CVC of the system, is represented with the black dashed line. The ratio of fatty acid to its respective glycerol monoester and/or alcohol was maintained at 2:1. UDA, undecylenic acid; UDG, glyceryl 1-undecylenate; UDOH, undecylenyl alcohol. n = 3; error bars represent standard deviation (s.d.).

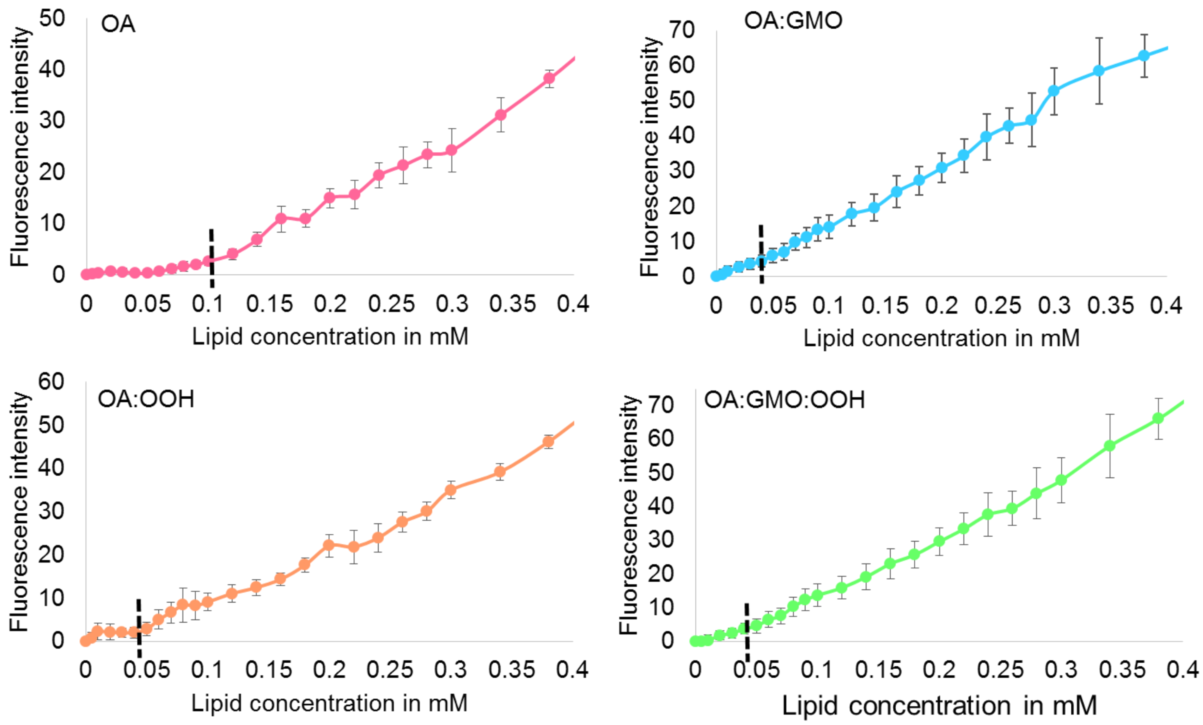

Figure S8: CVC estimation of the different C18 based membrane systems by fluorescence assay.

The increase in fluorescence is plotted as a function of total lipid concentration (fatty acid and derivatives). The inflection point, which is a read out of the CVC of the system, is represented with the black dashed line. The ratio of fatty acid to its respective glycerol monoester and/or alcohol was maintained at 2:1. OA, oleic acid; GMO, glycerol 1-monooleate; OOH, oleyl alcohol. n = 3; error bars represent standard deviation (s.d.).

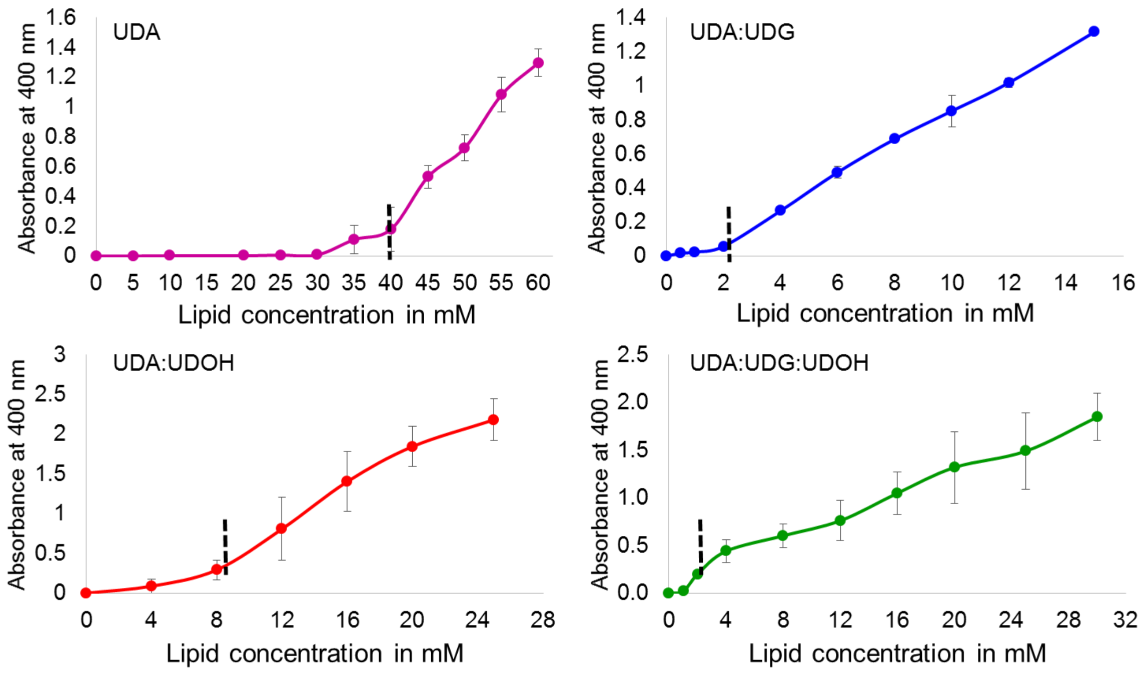

Figure S9: CVC estimation of C11 based systems by measuring absorbance at 400 nm.

The increase in absorption (at 400 nm) is plotted as a function of total lipid concentration (fatty acid and derivatives). The concentration of lipid when sudden increase in absorption (scattering) is observed is considered as the inflection point, indicated by black dashed vertical line. The ratio of fatty acid to its respective glycerol monoester and/or alcohol was maintained at 2:1. UDA, undecylenic acid; UDG, glyceryl 1-undecylenate; UDOH, undecylenyl alcohol. n = 3; error bars represent standard deviation (s.d.).

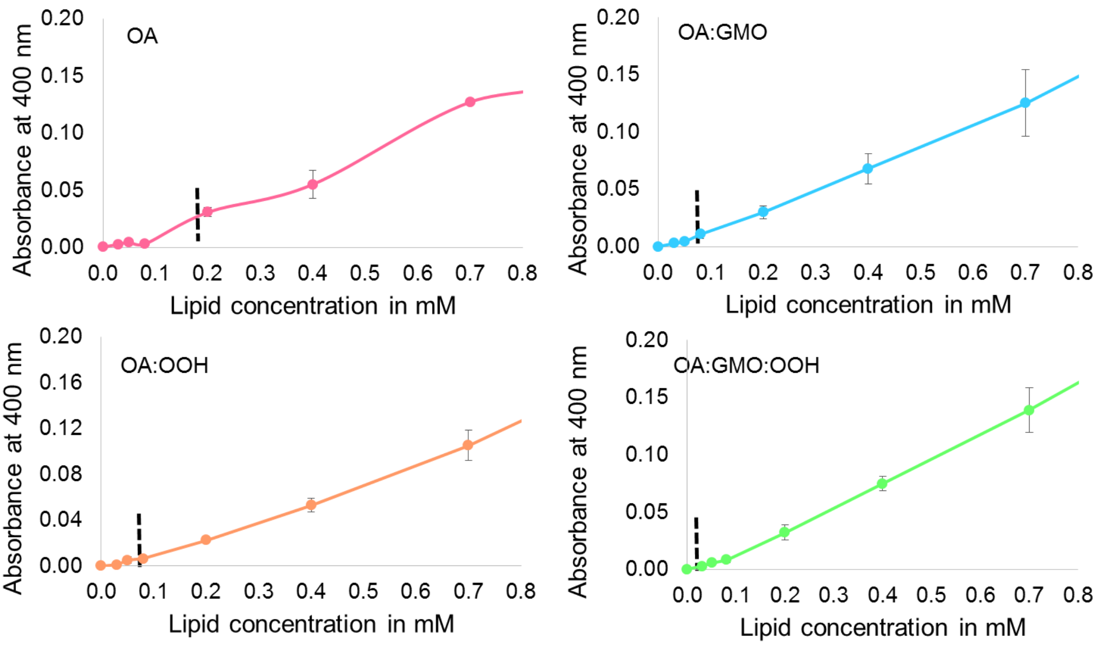

Figure S10: CVC estimation of different C18 based systems by measuring absorbance at 400 nm.

The increase in absorbance (scattering) is plotted as a function of total lipid concentration (fatty acid and derivatives). The concentration of lipid when sudden increase in absorbance is observed is considered as the inflection point, indicated by black dashed vertical line. The ratio of fatty acid to its respective glycerol monoester and/or alcohol was maintained at 2:1. OA, oleic acid; GMO, glycerol 1-monooleate; OOH, oleyl alcohol. n = 3; error bars represent standard deviation (s.d.).

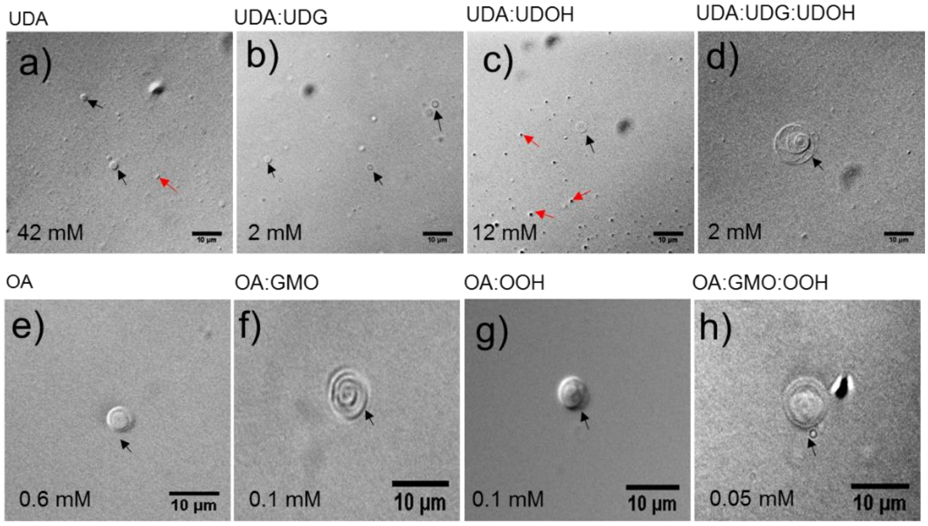

Figure S11: CVC estimation using microscopic analysis.

Panels a to d and e to f show the microscopic analysis of the different C11 and C18 based systems, respectively, at their CVC. a) 40 mM homogenous UDA, b) 2 mM of UDA:UDG, c) 12 mM of UDA:UDOH, d) 2 mM of the tertiary UDA:UDG:UDOH system, e) 0.6 mM of OA, f) 0.1 mM of OA:GMO, g) 0.1 mM of OA:UDOH, and h) 0.05 mM of OA:GMO:OOH systems. Below the aforesaid concentrations, vesicles were not observed under 40X magnification. The black and red arrows indicate vesicles and oil droplets, respectively. The ratio of fatty acid to its respective glycerol monoester and/or alcohol was maintained at 2:1. n = 3. Scale bar in all the images is 10 microns. UDA, undecylenic acid; UDG, glyceryl 1-undecylenate; UDOH, undecylenyl alcohol; OA, oleic acid; GMO, glycerol 1-monooleate; OOH, oleyl alcohol.

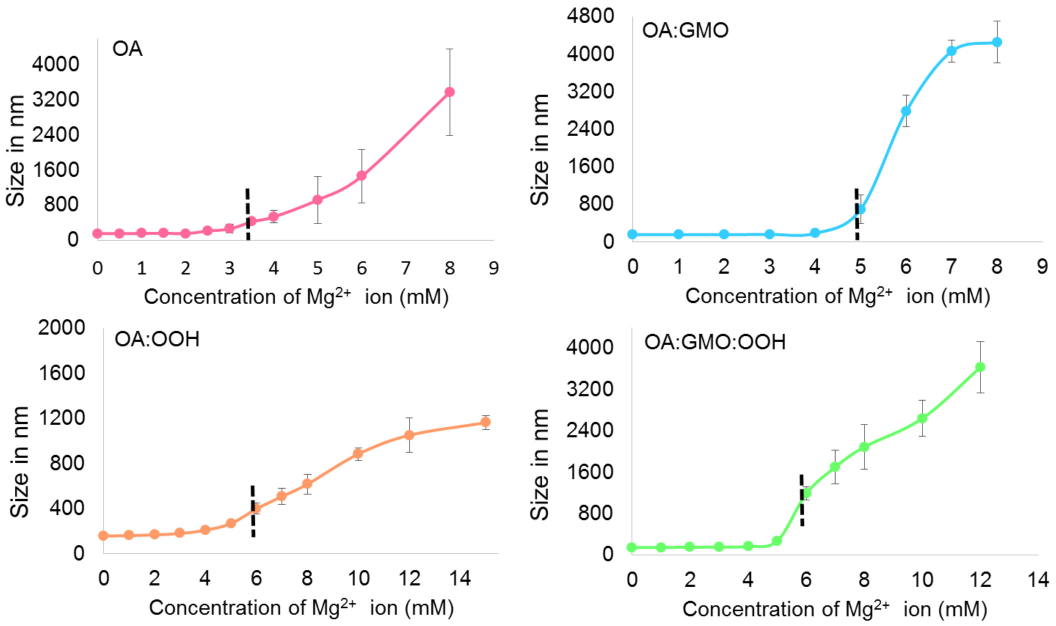

Figure S12: Membrane stability in presence of Mg2^{+} ions.

Shows DLS measurements of the C18 based membrane systems to determine Mg^{2+} ion induced aggregate (crystalline aggregates and collapsed vesicles) formation concentration. The particle diameter (in nm) is plotted as a function of the added Mg^{2+} ion concentration. The vertical black dashed line indicates the Mg^{2+} ion induce aggregation formation concentration ($Mg^{2+}{}_{AIC}$). The ratio of fatty acid to its respective glycerol monoester and/or alcohol was maintained at 2:1. n = 3; error bars represent standard deviation (s.d.). OA, oleic acid; GMO, glycerol 1-monooleate; OOH, oleyl alcohol.

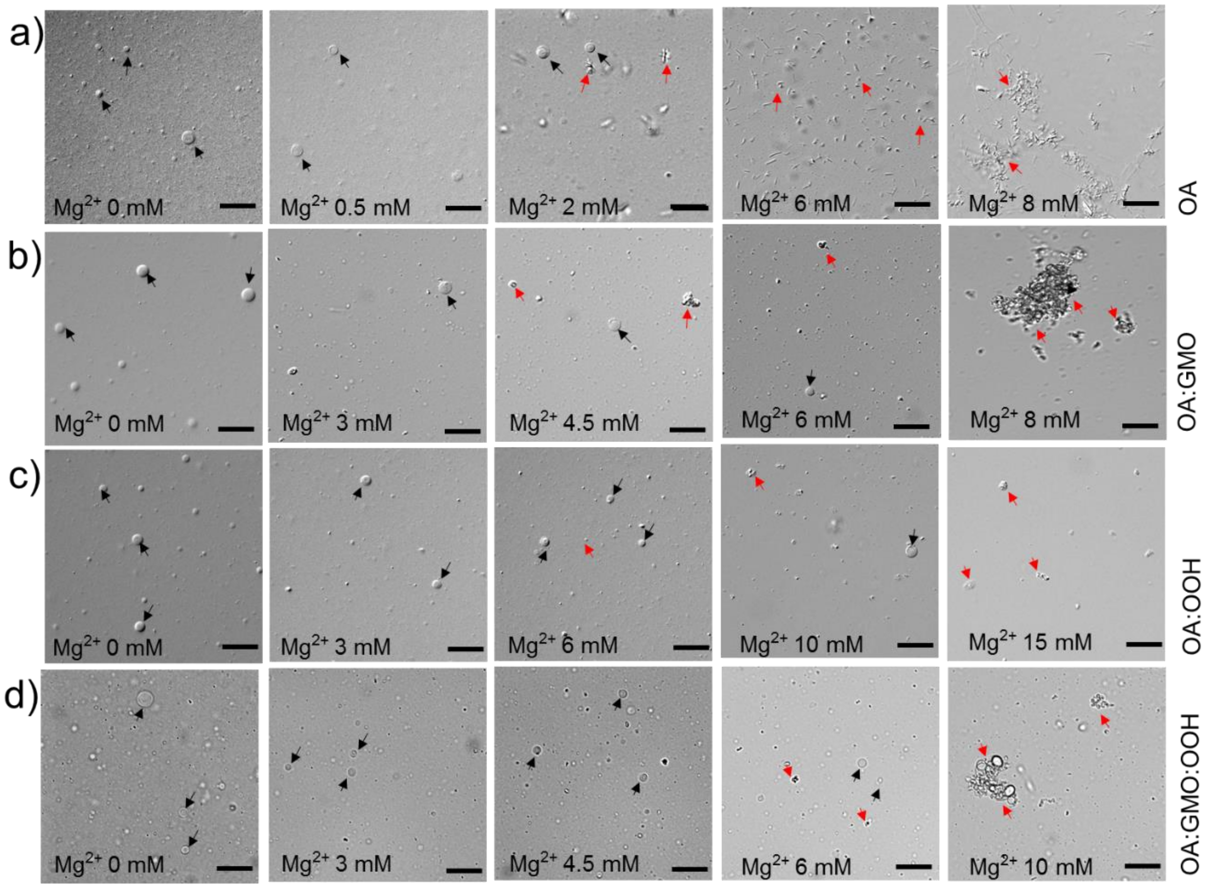

Figure S13: Microscopic analysis of C18 based membrane stability in presence of $Mg2^{+}$ ions.

Mg^{2+} ion induced aggregate forming properties of all the four C18 based membrane systems (panels a to d). From left to right, Mg^{2+} ion concentration was increased gradually by keeping the lipid concentration constant. The ratio of fatty acid to its respective glycerol monoester and/or alcohol was maintained at 2:1. In terms of cation sensitivity among the four systems, the following order is observed: OA > OA:GMO > OA:GMO:OOH = OA:OOH. The black and red arrows indicate vesicles and aggregates (fatty acid crystals and collapsed vesicles), respectively. The scale bar in all images is 20 microns. OA, oleic acid; GMO, glycerol 1-monooleate; OOH, oleyl alcohol.

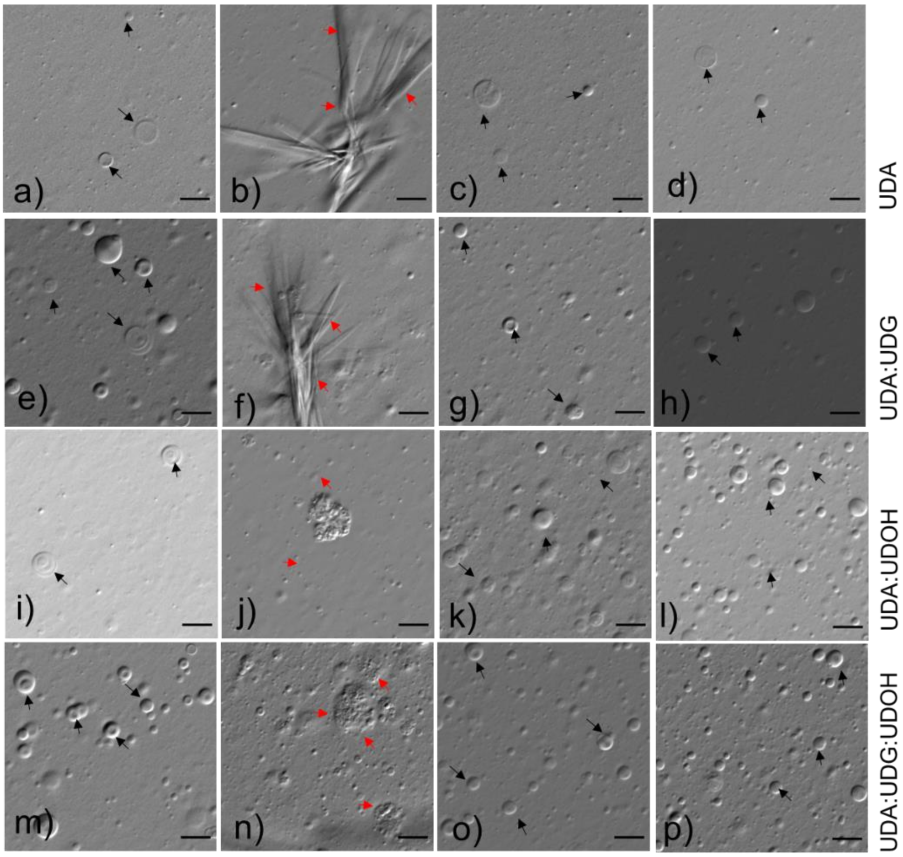

Figure S14: Reformation of vesicles form Mg^{2+} ion induced aggregates (crystalline aggregates and collapsed vesicles) using EDTA.

a) to d) indicates pure UDA systems. a) 60 mM pure UDA suspension; b) 60 mM UDA in presence of 4 mM Mg^{2+} ions; c) 60 mM UDA in presence of 4 mM Mg^{2+} ions and 4 mM EDTA and d) 60 mM UDA in presence of 4 mM Mg^{2+} ions and 8 mM EDTA. e) to h) indicates UDA:UDG (2:1) mixed systems. e) 60 mM UDA:UDG (2:1) mixed system; f) 60 mM UDA:UDG (2:1) in presence of 24 mM Mg^{2+} ions; g) 60 mM UDA:UDG (2:1) system in presence of 24 mM Mg^{2+} ions and 24 mM EDTA and h) 60 mM UDA:UDG (2:1) system in presence of 24 mM Mg^{2+} ions and 48 mM EDTA. i) to l) indicates UDA:UDOH (2:1) mixed systems, i) 60 mM UDA:UDOH (2:1) mixed system; j) 60 mM UDA:UDOH (2:1) system; in presence of 14 mM Mg^{2+} ions; k) 60 mM UDA:UDOH (2:1) system in presence of 14 mM Mg^{2+} ions and 14 mM EDTA and l) 60 mM UDA:UDOH (2:1) system in presence of 14 mM Mg^{2+} ions and 28 mM

EDTA. m) to p) indicates UDA:UDG:UDOH mixed tertiary systems, m) 60 mM UDA:UDG:UDOH (4:1:1) mixed system; n) 60 mM UDA:UDG:UDOH (4:1:1) in presence of 16 mM Mg^{2+} ions; o) 60 mM UDA:UDG:UDOH (4:1:1) system in presence of 16 mM Mg^{2+} ions and 16 mM EDTA and p) 60 mM UDA:UDG:UDOH (4:1:1) system in presence of 16 mM Mg^{2+} ions and 32 mM EDTA. The ratio of fatty acid to its respective glycerol monoester and/or alcohol was maintained at 2:1. The black and red arrows indicate vesicles and aggregates (Mg-soap crystal and collapsed vesicles), respectively. The scale bar in all the images is 10 microns.

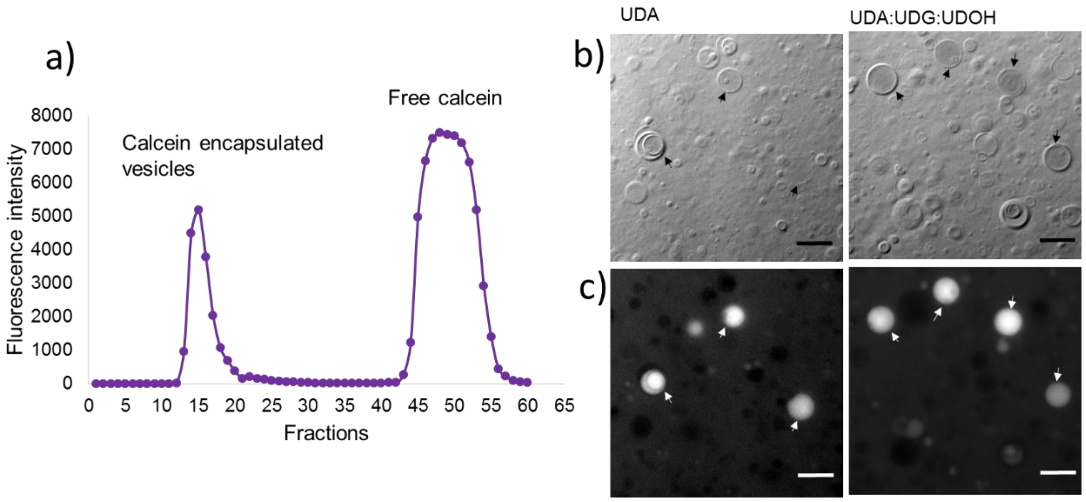

Figure S15: Calcein encapsulation and size size exclusion column chromatography. Panel a shows the size exclusion column chromatography profile, which was used to separate vesicles with encapsulated calcein, from the unencapsulated calcein. Fluorescence at 518 nm was plotted against the fraction number. The unencapsulated calcein comes out in later fractions whereas the vesicles with encapsulated calcein elute out in the earlier fractions. Panel b shows the epifluorescence micrographs of calcein encapsulated vesicles from two C11 based membrane systems. The images on the top represent the Differential Interference Contrast (DIC) images, while the lower two images are of the fluorescence images of the same field of view. The scale bar in all the images is 10 microns. UDA, undecylenic acid; UDG, glyceryl 1-undecylenate; UDOH, undecylenyl alcohol

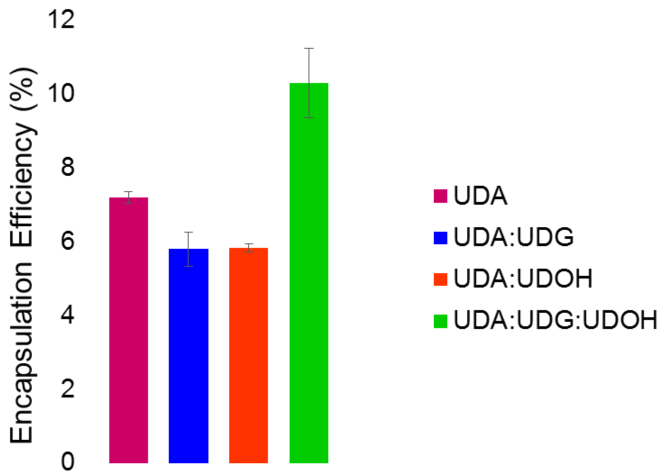

Figure S16: Estimation of encapsulation efficiency.

Estimation of encapsulation efficiency of four different C11 based membrane systems. Calcein was encapsulated in the vesicles. The experiment was performed in replicate. Error bars represent standard deviation (s.d.). The ratio of fatty acid to its respective glycerol monoester and/or alcohol was maintained at 2:1. UDA, undecylenic acid; UDG, glyceryl 1-undecylenate; UDOH, undecylenyl alcohol.

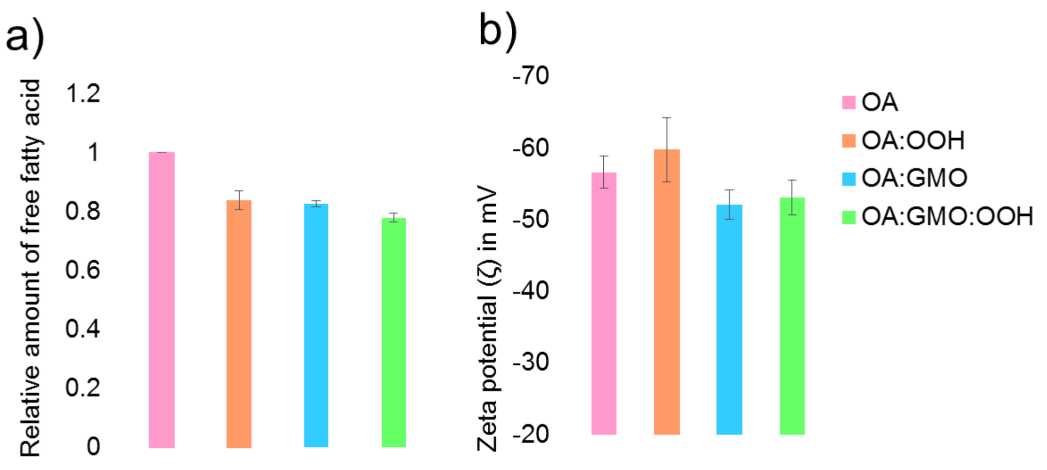

Figure S17: LC-MS analysis for free acid quantification and zeta potential measurements.

Panel a) represents the relative amount of free oleic acid molecules (C18) present in the suspension as a function of membrane composition in all four C18 based membrane systems. n = 6; error bars represent standard deviation (s.d.). The difference between the means for homogenous OA and the other three heterogeneous systems is significant based on student t test with a *p*-value < 0.05 (using a one-tailed test). Panel b represents the zeta potential measurements of the C18 based systems as a function of their composition. n = 5; error bars represent standard deviation (s.d.). The difference between the means of the OA system and that of the OA:GMO/ OA:GMO:OOH systems is significant based on student t test; with a *p*-< 0.05. The difference between the means obtained for the homogenous OA and the binary OA:OOH is not significant based on student t test: *p*-value > 0.05 (using a one-tailed test). OA, oleic acid; GMO, glycerol 1-monooleate; OOH, oleyl alcohol.

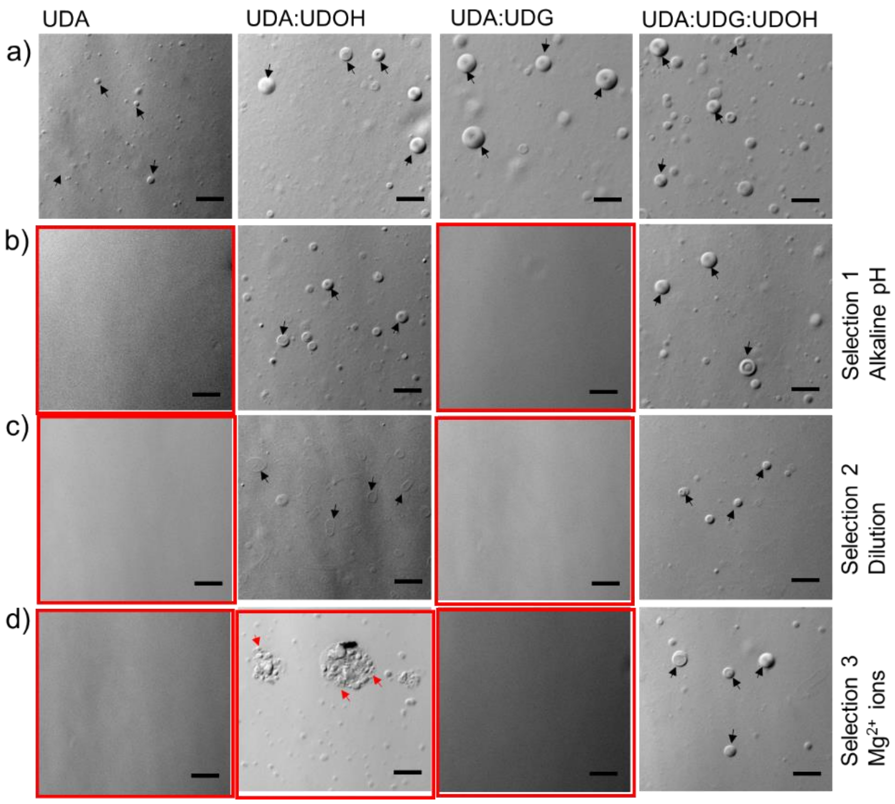

Figure S18: Vesicle stability under multiple selection pressures (MSPs).

Panels a to d represent the different pebiotically relevant selection conditions. a) All four C11 based systems at a concentration of 60 mM at pH 8. b) Stability at alkaline pH as a selection pressure. All systems comprised of 60 mM of lipid concentration, with the pH of the system now adjusted to 10. c) Dilution regime as selection pressure in which all the four systems were diluted to concentration of 20 mM lipid concentration at pH 10. d) Stability in the presence of Mg^{2+} ions as selection pressure. Mg^{2+} ions were added in all the systems at a concentration of 14 mM. The lipid concentration is 20 mM in all the systems, which are at pH10. The red boxes indicate conditions where the vesicles are absent. The black and red arrows indicate vesicles and aggregates (crystalline aggregates and collapsed vesicles), respectively. The scale bar in all the images is 10 microns.

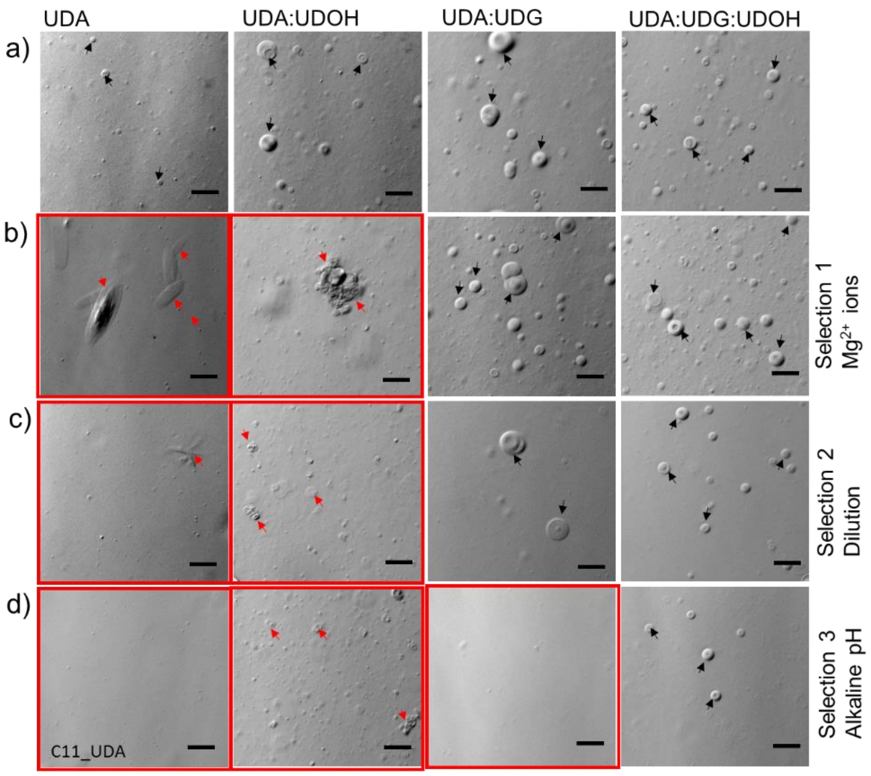

Figure S19: Vesicle stability under multiple selection pressures (MSPs).

Panels b to d represent the different pebiotically relevant selection conditions. a) All four C11 based systems at a concentration of 60 mM at pH 8. b) Stability in the presence of Mg^{2+} ions as selection pressure. Mg^{2+} ions were added in all the systems at a concentration of 14 mM. The lipid concentration is 60 mM in all the systems, which are at pH8. c) Dilution regime as selection pressure in which all the four systems were diluted to concentration of 20 mM lipid concentration at pH 8, in presence of 14 mM Mg^{2+} ions. d) Stability at alkaline pH as a selection pressure. All systems comprised of 20 mM of lipid concentration containing 14 mM Mg^{2+} ions, with the pH of the system now adjusted to 10. The red boxes indicate conditions where the vesicles are absent. The black and red arrows indicate vesicles and aggregates (crystalline aggregates and collapsed vesicles), respectively. The scale bar in all the images is 10 microns.

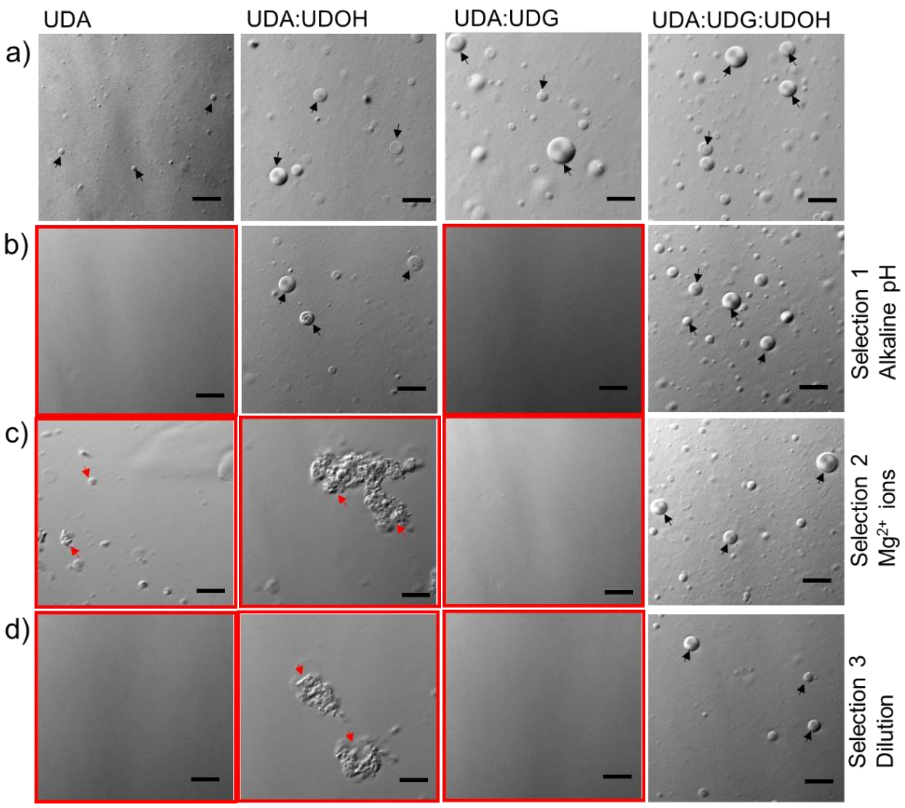

Figure S20: Vesicle stability under multiple selection pressures (MSPs).

Panels b to d represent the different pebiotically relevant selection conditions. a) All four C11 based systems at a concentration of 60 mM at pH 8. b) Stability at alkaline pH as a selection pressure. All systems comprised of 60 mM of lipid concentration, with the pH of the system now adjusted to 10. c) Stability in the presence of Mg^{2+} ions as selection pressure. Mg^{2+} ions were added in all the systems at a concentration of 14 mM. The lipid concentration is 60 mM in all the systems, which are at pH 10. d) Dilution regime as selection pressure in which all the four systems were diluted to concentration of 20 mM lipid concentration at pH 10 in presence of 14 mM Mg^{2+} ions. The red boxes indicate conditions where the vesicles are absent. The black and red arrows indicate vesicles and aggregates (crystalline aggregates and collapsed vesicles), respectively. The scale bar in all the images is 10 microns.

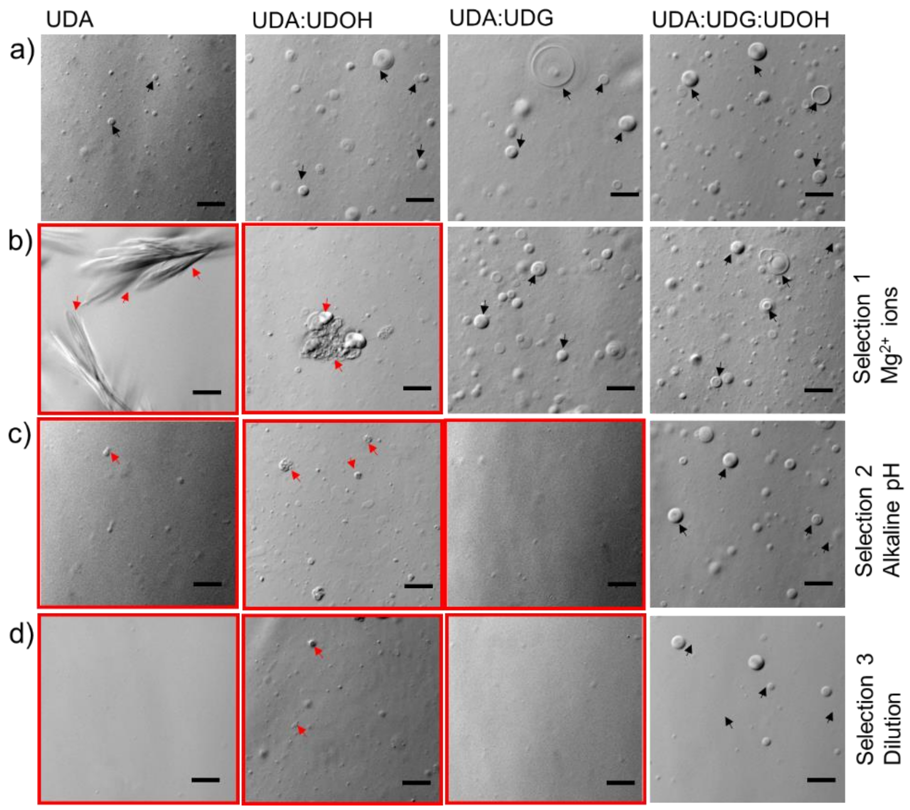

Figure S21: Vesicle stability under multiple selection pressures (MSPs).

Panels b to d represent the different pebiotically relevant selection conditions. a) All four C11 based systems at a concentration of 60 mM at pH 8. b) Stability in the presence of Mg^{2+} ions as selection pressure. Mg^{2+} ions were added in all the systems at a concentration of 14 mM. The lipid concentration is 60 mM in all the systems, which are at pH8. c) Stability at alkaline pH as a selection pressure. All systems comprised of 60 mM of lipid concentration and 14 mM Mg^{2+} ions, with the pH of the system now adjusted to 10. d) Dilution regime as selection pressure in which all the four systems were diluted to concentration of 20 mM lipid concentration at pH 10, in presence of 14 mM Mg^{2+} ions. The red boxes indicate conditions where the vesicles are absent. The black and red arrows indicate vesicles and aggregates (crystalline aggregates and collapsed vesicles), respectively. The scale bar in all the images is 10 microns.

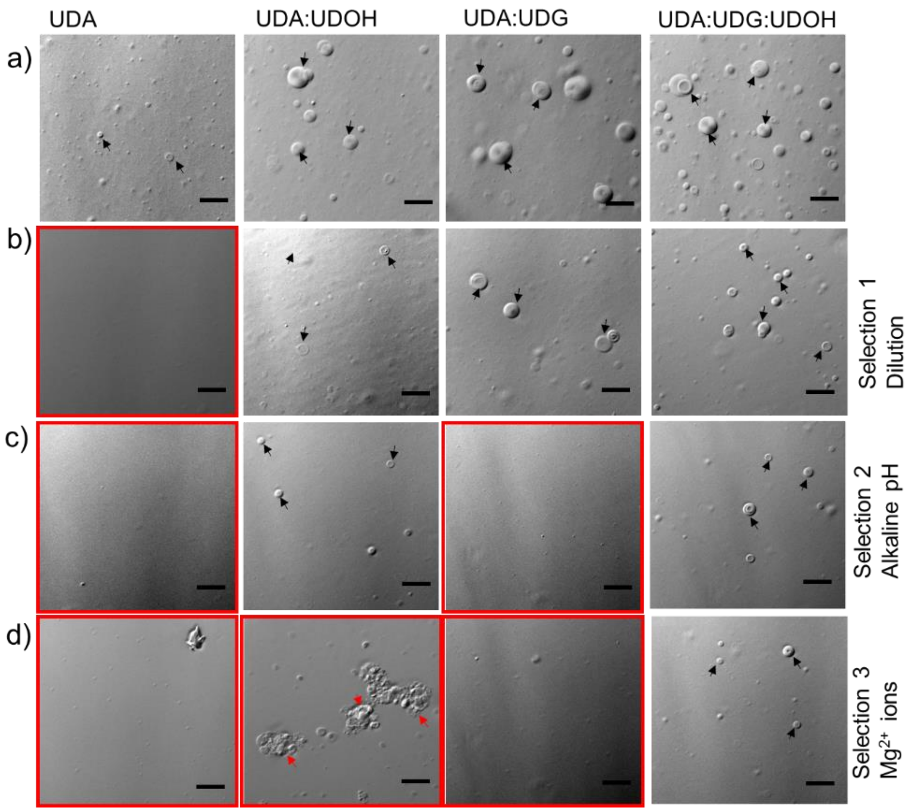

Figure S22: Vesicle stability under multiple selection pressures (MSPs).

Panels b to d represent the different pebiotically relevant selection conditions. a) All four C11 based systems at a concentration of 60 mM at pH 8. b) Dilution regime as selection pressure in which all the four systems were diluted to concentration of 20 mM lipid concentration at pH 8. c) Stability at alkaline pH as a selection pressure. All systems comprised of 20 mM of lipid concentration, with the pH of the system now adjusted to 10. d) Stability in the presence of Mg^{2+} ions as selection pressure. Mg^{2+} ions were added in all the systems at a concentration of 14 mM. The lipid concentration is 20 mM in all the systems, which are at pH10. The red boxes indicate conditions where the vesicles are absent. The black and red arrows indicate vesicles and aggregates (crystalline aggregates and collapsed vesicles), respectively.

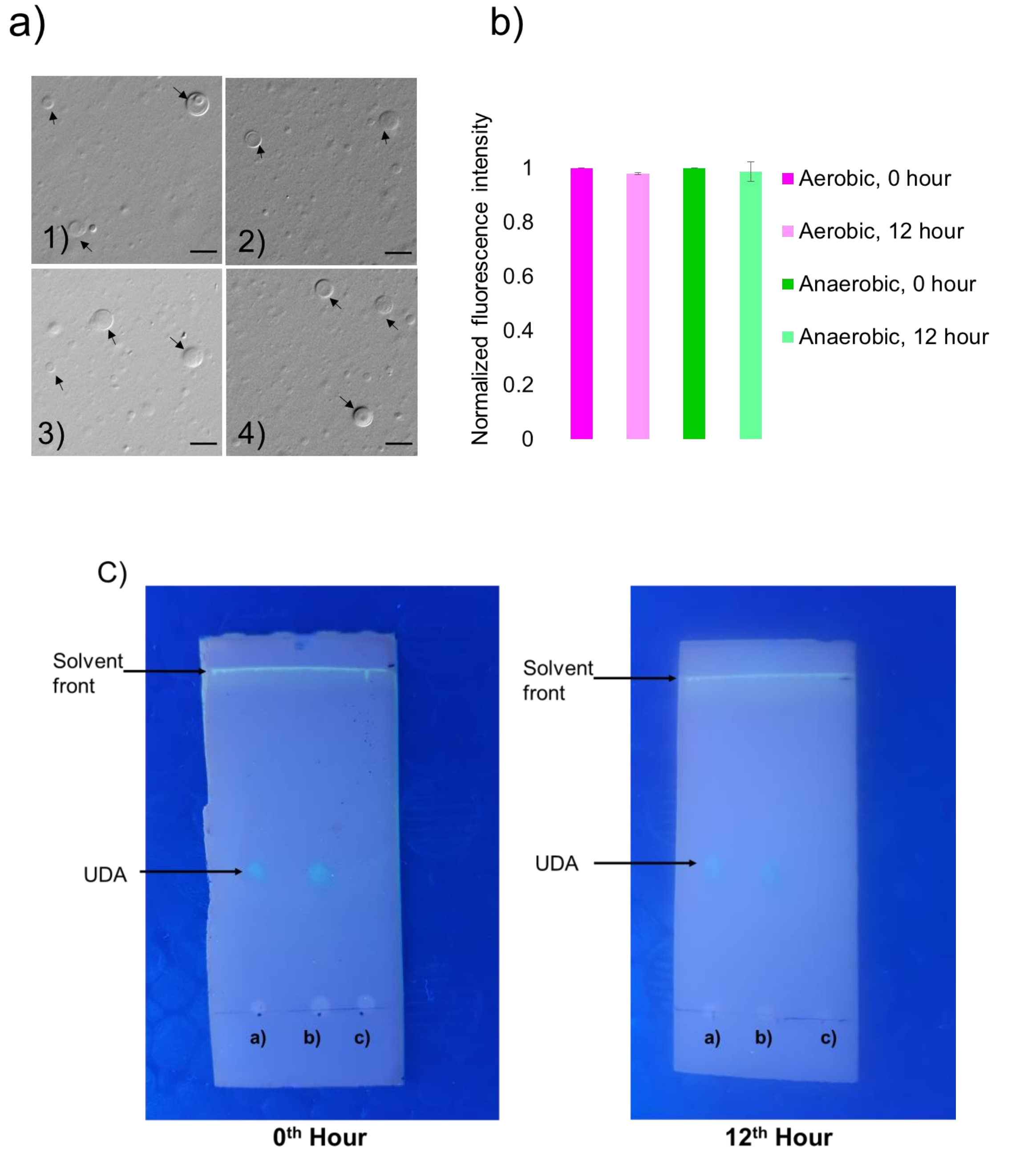

Figure S23: UDA oxidation stability.

In panel a), 1) to 4) shows the microscopic analysis of UDA membrane systems in anaerobic and aerobic condition at different time points. 1) 60 mM UDA system in aerobic condition at 0th hour; 2) 60 mM UDA system in aerobic condition at 12th hour; 3) 60 mM UDA system in anaerobic condition at 0th hour and 4) 60 mM UDA system

in anaerobic condition at 12th hour. The black arrows indicate vesicles. The scale bar in all the images is 10 microns.

Panel b) shows the fluorescence intensity which is a readout of the amount of bilayer UDA membrane systems in anaerobic and aerobic condition at different time points. The fluorescence of the 0th hour was normalized to one. The experiment was performed in replicate. Error bars represent standard deviation (s.d.).

Panel c) shows that qualitative TLC analysis of UDA membrane systems in anaerobic and aerobic condition at different time points. 1) to 3) indicates different lanes, 1) 60 mM mM UDA system in aerobic condition; 2) 60 mM mM UDA system in anaerobic condition and 3) 200 mM bicine buffer as the UDA suspension was prepared in 200 mM bicine buffer. After 12 hours of incubation no new band of oxidative degradation product was observed. The two TLC plates (uncropped) were imaged separately at different time points (with a 12 hours interval in between) using a handheld UV-light at 360 nm. The images were accrued using a smart phone camera. The two TLC plate images (without contrast adjust) have been grouped together in their entirety in the panel c).
